# Gene loss, adaptive evolution and the co-evolution of plumage coloration genes with opsins in birds

**DOI:** 10.1186/s12864-015-1924-3

**Published:** 2015-10-06

**Authors:** Rui Borges, Imran Khan, Warren E. Johnson, M. Thomas P. Gilbert, Guojie Zhang, Erich D. Jarvis, Stephen J. O’Brien, Agostinho Antunes

**Affiliations:** CIIMAR/CIMAR, Interdisciplinary Centre of Marine and Environmental Research, University of Porto, Rua dos Bragas, 177, 4050-123 Porto, Portugal; Department of Biology, Faculty of Sciences, University of Porto, Rua do Campo Alegre, 4169-007 Porto, Portugal; Smithsonian Conservation Biology Institute, National Zoological Park, 1500 Remount Road, Front Royal, VA 22630 USA; Centre for GeoGenetics, Natural History Museum of Denmark, University of Copenhagen, Øster Volgade 5-7, 1350 Copenhagen, Denmark; China National GeneBank, BGI-Shenzhen, Shenzen, 518083 China; Centre for Social Evolution, Department of Biology, Universitetsparken 15, University of Copenhagen, DK-2100 Copenhagen, Denmark; Department of Neurobiology, Duke University Medical Center Durham, Box 3209, North Carolina, 27710 USA; Howard Hughes Medical Institute, Chevy Chase, Maryland, 20815 USA; Theodosius Dobzhansky Center for Genome Bioinformatics, St. Petersburg State University, St. Petersburg, 199004 Russia; Oceanographic Center, Nova Southeastern University, 8000 N. Ocean Drive, Ft Lauderdale, Florida, 33004 USA

**Keywords:** Opsin, Vision, Birds, Gene loss, Pseudogenization, Co-evolution, Plumage coloration

## Abstract

**Background:**

The wide range of complex photic systems observed in birds exemplifies one of their key evolutionary adaptions, a well-developed visual system. However, genomic approaches have yet to be used to disentangle the evolutionary mechanisms that govern evolution of avian visual systems.

**Results:**

We performed comparative genomic analyses across 48 avian genomes that span extant bird phylogenetic diversity to assess evolutionary changes in the 17 representatives of the opsin gene family and five plumage coloration genes. Our analyses suggest modern birds have maintained a repertoire of up to 15 opsins. Synteny analyses indicate that *PARA* and *PARIE* pineal opsins were lost, probably in conjunction with the degeneration of the parietal organ. Eleven of the 15 avian opsins evolved in a non-neutral pattern, confirming the adaptive importance of vision in birds. Visual conopsins *sw1*, *sw2* and *lw* evolved under negative selection, while the dim-light *RH1* photopigment diversified. The evolutionary patterns of *sw1* and of violet/ultraviolet sensitivity in birds suggest that avian ancestors had violet-sensitive vision. Additionally, we demonstrate an adaptive association between the *RH2* opsin and the *MC1R* plumage color gene, suggesting that plumage coloration has been photic mediated. At the intra-avian level we observed some unique adaptive patterns. For example, barn owl showed early signs of pseudogenization in *RH2*, perhaps in response to nocturnal behavior, and penguins had amino acid deletions in *RH2* sites responsible for the red shift and retinal binding. These patterns in the barn owl and penguins were convergent with adaptive strategies in nocturnal and aquatic mammals, respectively.

**Conclusions:**

We conclude that birds have evolved diverse opsin adaptations through gene loss, adaptive selection and coevolution with plumage coloration, and that differentiated selective patterns at the species level suggest novel photic pressures to influence evolutionary patterns of more-recent lineages.

**Electronic supplementary material:**

The online version of this article (doi:10.1186/s12864-015-1924-3) contains supplementary material, which is available to authorized users.

## Background

Birds are highly visual animals, with a variety of special adaptations to diverse light stimuli [1–3]. Anatomical, physiological, genetic and paleontological evidence suggests that birds rely heavily on visual cues in most aspects of their life history [1]. The bird’s eye occupies around 50 % of the cranial volume while retaining the same general structure as other vertebrates [1]. The eye has an ellipsoid conformation, a sclerotic ring that integrates a large visual field and a specialized retina that provides high focal acuity [1]. Birds possess cone photoreceptors that are distributed densely over the retina (i.e. multiple foveas) [2] and generally possess a tetrachromatic visual system, with colored oil droplets containing a high concentration of carotenoids that are associated with cone cells [1, 3], acting as filters that enhances color discrimination [4]. In contrast, most mammals have two types of cone opsins and are thus generally dichromatic [3].

Avian eye complexity has led to a complex array of ecological visual specializations, including use of ultra-violet sensitivity (UVS) [5]. Birds employ UVS variation broadly in recognition of coloration patterns, social signaling, hunting, nectar localization and mate-choice [6–8]. Color cues are also important in avian intra-specific and inter-specific communication, including for evaluating the quality of potential mates (sexual selection), select resources, spot elusive prey and detect predators. Furthermore, birds can use photic stimuli in spatio-temporal orientation. For example, some species use a refined sense of photoperiodicity and day length to assist in magnetic compass orientation [9] and regulate seasonal behaviors including sexual periods and seasonal migratory patterns [10]. Although photoreception is a crucial and ubiquitous trait of birds, our understanding of the co-evolution of behaviors and patterns of genetic variation underlying avian photoreception remains poor.

Birds share with all vertebrates a general mechanism of photoreception that is mediated by opsins, a group of hepta-transmembrane proteins [11] involved in the conversion of a photon of light into an electrochemical signal. Vertebrate opsins have been phylogenetically classified into five subfamilies: (1) the visual opsins, including rhodopsin (*RH1*) and conopsins (*RH2*, *OPN1sw1*, *OPN1sw2* and *OPN1lw*); (2) the melanopsins consisting of two paralogous genes (*OPN4m* and *OPN4x*); (3) the pineal subfamily consisting of the parapinopsin (*PARA*), parietopsin (*PARE*), pinopsin (*PIN*) and vertebrate ancient (*VA*) opsins; (4) the vertebrate non-visual subfamily including encephalopsin (*OPN3*) and the teleost multiple tissue opsins (*TMT* and *TMT2*); and (5) a photoisomerases group including the *RGR*, *RRH* and neuropsin (*OPN5*) genes [11, 12]. These last three groups are referred *sensu lato* as the non-visual opsins, as they are involved in non-image forming responses to light [13].

In a preliminary companion study, we found that opsin genes show evidence of having evolved under strong stabilizing selection in birds, with mean ω values below 0.25 [14]. However, some episodes of positive selection were identified in the *RH2* and *OPN1sw1* opsins on the emerging branches of penguins and Passerida [14]. Among the visual opsins, *OPN1sw1* plays a role in avian sensitivity to violet (VS) or UVS light, with evidence of positive selection in Passerida probably related with the spectral tuning change from UVS-VS. These shifts can be explained by single nucleotide substitutions at the 86 and 90 spectral tuning sites found in the partial sequence of *OPN1sw1* across birds in a past study [15]. Similarly, there is phylogenetic evidence suggesting that avian color vision has shifted between VS and UVS at least 14 times within avian evolution [16].

Despite efforts to better understand the evolution of opsin in birds, most of these studies have focused on visual opsins, and PCR generated fragments of the genes. However, non-visual responses are as important as visual for the overall light adaptive response, and a more complete study is needed to better elucidate the biological details of avian photic adaptation. Here we advanced our findings by performing a comparative analyses across 48 avian species, most recently sequenced [14, 17], to characterize gene gain/loss and the selective forces that have occurred in the 17 vertebrate representatives of the opsin gene family. Our findings provide insight into the history of avian photic adaptation and their co-evolving systems.

## Results

### Genomic identification of avian opsins

**Fig. 1:**
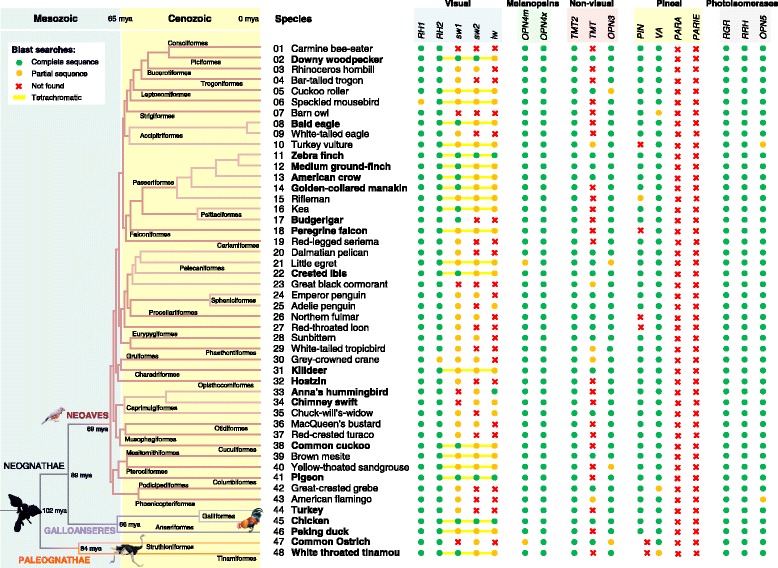
The presence/absence patterns of avian opsins. Green circles indicate the presence of a complete gene sequence; yellow circles represent a partial gene sequence; red cross indicates that no sequences were found by t-blastn searches. For the visual opsins, the species highlighted with a yellow line have a tetrachromatic visual system. The bird phylogeny and the mean divergence times were based on Jarvis *et. al* (2014) [17]. The high coverage genomes (≥80X) are indicated in bold. Numbers identify each species: 1. *Merops nubicus*, 2. *Picoides pubescens*, 3. *Buceros rhinoceros*, 4. *Apaloderma vittatum*, 5. *Leptosomus discolor*, 6. *Colius striatus*, 7. *Tyto alba*, 8. *Haliaeetus leucocephalus*, 9. *Haliaeetus albicilla*, 10. *Cathartes aura*, 11. *Taeniopygia guttata*, 12. *Geospiza fortis*, 13. *Corvus brachyrhynchos*, 14. *Manacus vitellinus*, 15. *Acanthisitta chloris*, 16. *Nestor notabilis*, 17. *Melopsittacus undulatus*, 18. *Falco peregrinus*, 19. *Cariama cristata*, 20. *Pelecanus crispus*, 21. *Egretta garzetta*, 22. *Nipponia nippon*, 23. *Phalacrocorax carbo*, 24. *Aptenodytes forsteri*, 25. *Pygoscelis adeliae*, 26. *Fulmarus glacialis*, 27. *Gavia stellata*, 28. *Eurypyga helias*, 29. *Phaethon lepturus*, 30. *Balearica regulorum*, 31. *Charadrius vociferus*, 32. *Opisthocomus hoazin*, 33. *Calypte anna*, 34. *Chaetura pelagica*, 35. *Antrostomus carolinensis*, 36. *Chlamydotis macqueenii*, 37. *Tauraco erythrolophus*, 38. *Cuculus canorus*, 39. *Mesitornis unicolor*, 40. *Pterocles gutturalis*, 41. *Columba livia*, 42. *Podiceps cristatus*, 43. *Phoenicopterus ruber*, 44. *Meleagris gallopavo*, 45. *Gallus gallus*, 46. *Anas platyrhynchos*, 47. *Struthio camelus* and 48. *Tinamus major*

**Fig. 2:**
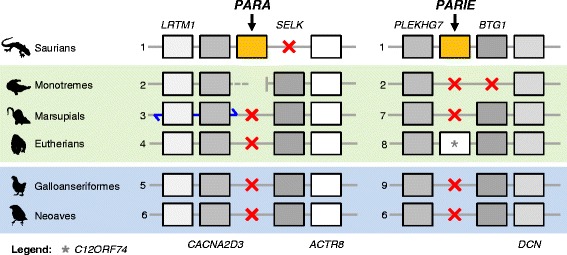
Syntenic patterns of the *PARA* and *PARIE* pineal opsins in mammals and birds. For each species we list the genes adjacent to *PARA* and *PARIE* and indicate when they are absent in genome sequences with a red cross. Blue arrow indicates that the region experienced an inversion. The numbers identify each species: 1. Carolina anole (*Anolis carolinensis*), 2. Platypus (*Ornithorhynchus anatinus*), 3. Tasmanian devil (*Sarcophilus harrisii*), 4. Human (*Homo sapiens*), 5. Chicken (*Gallus gallus*), 6. Zebra finch (*Taeniopygia guttata*), 7. Opossum (*Monodelphis domestica*), 8. African elephant (*Loxodonta africana*) and 9. Turkey (*Meleagris gallopavo*)

We retrieved sequences of opsin genes through t-blastn searches in 48 bird genomes [17] using the well annotated chicken (*Gallus gallus*) and zebra finch (*Taeniopygia guttata*) opsin gene sequences as queries. We found most opsins assumed to be present in tetrapoda genomes: *RH1*, *RH2*, *OPN1lw*, *OPN1sw1*, *OPN1sw2*, *OPN4m*, *OPN4x*, *OPN3*, *RGR*, *RRH*, *OPN5*, *PIN* and *VA* (Fig. 1). We also identified for the first time the two different types of teleost multiple tissue opsin genes in birds (designated as *TMT* and *TMT2*). We could not find the parietopsin and parapinopsin pineal opsins (*PARA* and *PARIE*) in any of the 48 studied genomes (blast searches conducted on the raw read sequences; Fig. 1). Syntenic analyses of other genes around where *PARA* and *PARIE* pineal genes were expected suggest that they were lost in birds and mammals (Fig. 2); only non-avian reptiles have the *PARIE* and *PARA* genes. These results suggest that birds have a repertoire of 15 opsin representatives, and that *PARIE* and *PARA* were independently lost in both birds and mammals.

The presence/absence patterns of opsins on the avian phylogenetic (evolutionary) tree [17] suggests no clear patterns of gene loss (or gain) among avian orders (Fig. 1). This is evidence that no major early events of gene loss (and gain) have occurred during the radiation of the avian lineages and evidence that the avian ancestor had 15 opsin representatives. This also supports the supposition that the tetrachromatic condition (lineages in which we were able to identify the *RH2*, *sw1*, *sw2* and *lw* visual conopsins) was the ancestral condition of modern birds.

*RH1* and *RH2* were present in all the bird genomes, while the *sw1*, *sw2* and *lw* conopsins were identified in 41, 31 and 26 of the 48 genomes, respectively (Fig. 1). Even in the high-coverage sequenced genomes (≥80X) we were unable to identify *sw1* in Anna's hummingbird (*Calypte anna*) and the closely related chimney swift (*Chaetura pelagica*), *lw* and *sw2* in hoatzin (*Opisthocomus hoazin*) and *lw* in the ostrich (*Strutio camelus*). However, experimental studies have shown that the ostrich possesses four types of cones [18] and *sw1* has been previously described in other Trochilidae (humingbirds) [19]. This suggests that some of these absences could be due to the inability to sequence these genes.

To assess if *sw1*, *sw2* and *lw* conopsins could be difficult to sequence genes, we assessed the GC (guanine-cytosine dinucleotide) content in all opsin genes using the GC ratio method (Additional file 1), which quantifies the relative abundance of the GC dinucleotide considering the abundance of each of the C and G nucleotides in the nucleotide sequence [20]. GC rich regions are known to be more difficult to sequence [21]. We found significant differences in the log GC ratio between opsin genes (ANOVA F = 77.67 on 14 and 559 degrees of freedom, *p*-value < 0.001), with *sw1*, *sw2* and *lw* conopsins having significantly higher GC ratios compared with all the other opsins (post-hoc pairwise comparisons, Additional file 2). This finding suggests that *sw1*, *sw2* and *lw* conopsins are located in GC-rich regions (such as the microchromosomes), which are known to be more difficult to sequence.

### Site-selection in avian and mammalian opsins

Using sequences retrieved from the 48 avian genomes we conducted phylogenetic and selection analyses. From the codon-based alignments and using the species phylogenetic tree (Fig. 1; [17]), we estimated the ω-ratio (the ratio between the non-synonymous by synonymous rate of substitutions) as an indicator of selective pressures acting on protein-coding genes [22]. Six of the 15 avian opsins, *RH1*, *PIN*, *VA*, *RGR*, *RRH* and *OPN4x*, showed evidence of positive selection (Table 1 and Additional file 3) while five, *OPN1sw1*, *OPN1sw2*, *OPN1lw*, *TMT* and *OPN3*, showed evidence of negative selection. *RH2*, *TMT2*, *OPN5* and *OPN4m* evolved neutrally. We found statistical evidence (>0.95 posterior probability) that 217A in *RH1* was positively selected (*G. gallus* amino acid number based on bovine rhodopsin amino acid numeration). We performed phylogenetic reconstruction of the 217 site using the well resolved avian species tree [17]. We found that 217 T/M/A residues are associated with the evolution of neoaves in water and land environments (Additional file 4). The land bird clades recurrently evolved the M residue, while the water bird clades evolved the T/A residues.

**Table 1:** Site-selection tests for the avian and mammalian opsins

Gene	ωB	ωM	E[*p*]	Odd score
*RH1*	0.061**	0.064*	0.411	1.429
*RH2*	0.080			
*OPN1sw1*	0.041*	0.178**	0.261	2.822
*OPN1sw2*	0.050*			
*OPN1lw*	0.024*	0.135	0.215	3.654
*TMT*	0.220*			
*TMT2*	0.118			
*OPN3*	0.101*	0.310**	0.210	3.761
*PIN*	0.230**			
*VA*	0.265**			
*RGR*	0.148**	0.193	0.316	2.166
*RRH*	0.155**	0.273**	0.333	2.000
*OPN5*	0.112	0.089	0.497	1.013
*OPN4x*	0.171**			
*OPN4m*	0.203	0.239**	0.470	1.128

ω values were calculated using the ω categories and the respective proportions under the statistcally significant site-selection model: (**) positive selection, (*) negative selection or neutral evolution (unmarked). *p* is the probability of a sampled ωB category being higher than a sampled ωM category. E[*p*] is the expected value of *p* using the bootstrap technique for 100 000 bootstraps. The odd score is the (1 - *p*)/*p* ratio and indicates the likelihood ωM categories are higher than the ωB categories

To test whether these changes are specific to birds, or present more broadly, we performed site-selection tests models on mammalian opsins. We found mammals possess 4 opsins evolving under positive selection (*sw1*, *OPN3*, *RRH* and *OPN4m*) and only one evolving through negative selection (*RH1*) (Table 1 and Additional file 3). We compared the ω-ratios in mammals and birds using the estimated ω-site categories (ωB and ωM) and their proportions under the statistically significant site-selection model. We used the bootstrap technique (10,000 replicates) to estimate the expected value of the probability of a sampled ωB category being higher than a sampled ωM category (*p*). The expected values of *p* and the respective odd scores (>2.5) indicate that mammals evolved with higher adaptive rates at the sites in *sw1*, *lw* and *OPN3* (2.82, 3.65, 3.77 odd score, respectively; Table 1).

### Species-specific branch selection

**Fig. 3:**
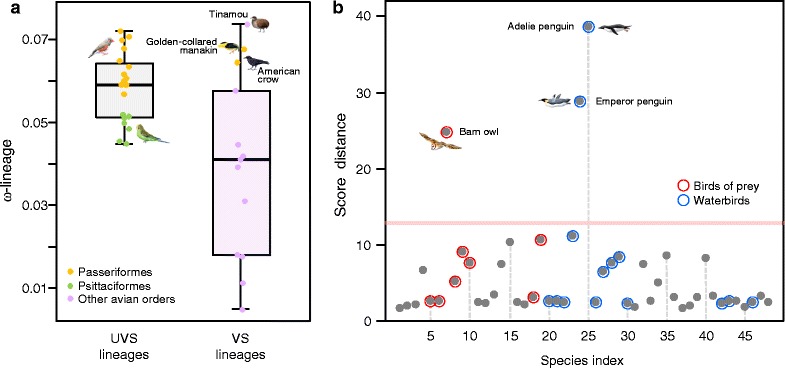
Lineage-specific visual adaptations in birds. **a** Box plots summarizing the lineage-specific evolutionary rates in bird species that possessed a violet (VS) or ultra-violet (UVS) *sws1* opsin. Colored circles identify the observed ω-lineage for Passeriformes (yellow) and Psittaciformes (green). **b** Distance plot indicating the lineages with an outlier evolutionary behavior in the opsin gene family. Species highlighted with red circumferences are birds of prey and those in blue are water birds. The species numbering system is the same as in Fig. 1

Branch-specific selection models implemented in PAML [22] were used to estimate the foreground evolutionary rate of each opsin gene in each of the studied species (ω-lineage) (Additional file 5). We implemented a phylogenetic logistic regression between the VS and UVS condition in each avian species and the respective ω-lineage value. Ödeen *et al.* (2009) have validated the use of genomic DNA to predict the VS and UVS subtypes in *sw1* opsins [23]. All the amino acid patterns we found in the 84–94 region of our sequences were already described in the literature [15, 16, 24], thus providing confirmation of VS/UVS inferences (Additional material 6). The boxplots (Fig. 3a) depict that *sw1* ω-lineage values are strongly correlated with the VS/UVS condition in birds, with the accelerated lineages being the most-strongly linked with UVS sensitivity: VS log-odd score = 4.44 – 79.91ω (*p*-value = 0.046; Wald z-statistics, Additional file 6). The VS log-odd score corresponds to the logarithm of the ratio between the probability of a certain lineage to be VS and its contrary (to be UVS) given the ω-lineage. The inferred ancestral condition for the most recent common ancestor of birds (ω-lineage = 0) was one of increased VS sensitivity (as opposed to UVS), with a 4.44 log-odd score (i.e. with 98.8 % of probability). In addition, the golden-collared manakin (*Manacus vitellinus*) and the American crow (*Corvus brachyrhynchos*) Passeriformes and the tinamou (*Tinamus major*) showed a relative higher ω-lineage for the VS-type (Fig. 3a).

We used principal component analysis with an outlier map to assess which lineages had unique patterns in opsins based on their ω-tendencies. The most informative principal components revealed prominent clusters consistent with the inferences gained from the phylogenetic hierarchy (i.e. closely related species were plotted nearby - results not shown). However, three species were clear outliers: the Adélie and emperor penguins (*Pygoscelis adeliae* and *Aptenodytes forsteri*) and the barn owl (*Tyto alba*) (Fig. 3b).

Sequence analysis of the barn owl *RH2* opsin, had several non-synonymous mutations in very conserved regions of the gene and indels and a stop codon at position 168 where the W residue is present in other all birds (Additional file 7). In penguins, the *RH2* sequence has a segmental deletion in the S295-S298 region. In addition, the *PIN* sequence in Adélie penguin had evidence of pseudogenization, with a frame-shift alteration and a stop codon. Li *et al.* (2014) suggested that there was a pseudogenization event for *PIN* in both penguins and reported several stop codons and frame shift alterations [25]. Although these features were found in the Adélie penguin, they were not observed in the emperor penguin *PIN* sequence (Afo_R013563). Further inspection of the penguins *RH1* amino acid sequences revealed a site-specific variation in the 194 tuning site [26]: L → P.

We additionally implemented branch-specific tests on the terminal lineage of the barn owl and the stem lineage of penguins to test whether the ω-ratio on these branches (ωf) have differentiated adaptive behavior (Additional file 8): in the barn owl lineage we found a significant accelerated ω-branch for *PIN* (ωf/ωb = 0.548/0.146); in penguins, *RH1* (0.119/0.043), *OPN4x* (0.546/0.155), *PIN* (0.527/0.146) and *OPN5* (0.408/0.107) also had a significantly accelerated ωf. Due to the early signs of pseudogenization in the barn owl *RH2* and the emperor penguin *PIN* opsins, we did not implement branch-specific tests for those lineages. We have also excluded the *TMT* and *VA* opsins from branch-tests of the in the barn owl lineages because only partial sequences were available (Fig. 1).

### Opsin *vs.* melanin-based coloration genes co-evolution analyses

In order to assess if the evolution of photoreception in birds is related with the evolution of plumage coloration, we also analyzed the melanin-based plumage coloration genes: *ASIP*, *OCA2*, *TYR*, *TYRP1* and *MC1R* [27]. These genes are part of the biochemical cascade leading to melanin production, which is, along with the carotenoids, responsible for pigmentation in birds [28, 29]. Site-selection analyses have shown that *MC1R* evolved under negative selection, while evidence of positive selection was found in the *OCA2*, *TYR* and *ASIP* (Additional material 3).

**Fig. 4:**
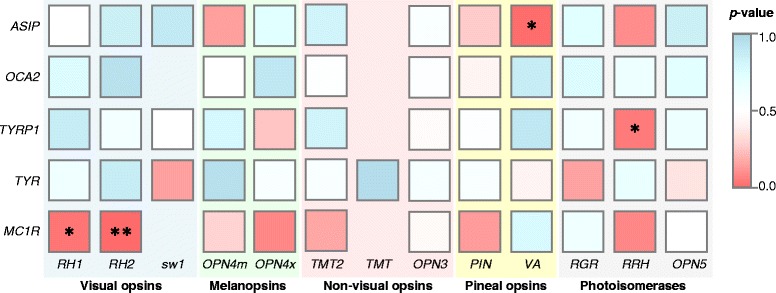
Co-evolution between opsins and the melanin-based plumage coloration genes in birds. Association tests were implemented using the ω-lineage classified as either: accelerated, conserved or neutral. The association between two genes was measured using the proportion of lineages showing the same evolutionary behavior and considering the alternative hypothesis *p* > 6/16. Significant (*p*-value < 8.4 x 10^−4^, **) and strong (*p*-value < 0.05, *) associations are indicated

To test for gene-evolution associations, we calculated the species-specific ω-lineage for the melanin-based plumage coloration genes, as we did for the avian opsin genes. We classified the ω-lineages into three evolutionary categories: accelerated (more than 0.75 quartile), conserved (less than the 0.25 quartile) and neutral, and implemented association tests for each of the visual-coloration gene pairs. The expected proportion of lineages with the same evolutionary category under independence was 6/16 (0.375), which was tested against the alternative hypothesis that *p* > 6/16. We found a significant association (*p*-value < 8.4e-4, Bonferroni corrected for multiple tests) between *MC1R* and *RH2* (Fig. 4; we excluded the *OPN1sws2* and *OPN1lw* opsins due to insufficient sampling size for implementing the association tests (≤15)). The statistical parameters of the co-evolution analysis are in Additional file 9. These associations are not related by physical location in the genome, as relative syntenic location analyses show that the associations are physically independent of genome location (Additional file 10).

## Discussion

We believe that our study is among the most comprehensive genomic analyses of opsins in vertebrates, and particularly in birds. We have been able to characterize losses, gains, and selective evolution that are correlated with lineage-specific traits. Below we discuss the implications of the key highlights for each family of opsins.

### The loss of *PARA* and *PARIE* in mammals and birds

The apparent independent loss of *PARA* and *PARIE* in mammals and birds is intriguing. In non-avian and non-mammalian vertebrates *PARA* and *PARIE* pineal opsins genes are expressed in the parietal organ, which is a part of the epithalamus [30]. The parietal organ, along with the pineal organ, forms the parietal eye (or third eye), which functions as a proper photoreception organ, regulating circadian rhythmicity and hormone production for thermoregulation [30]. In birds and mammals the parietal organ degenerates completely [12, 31, 32], which is very likely associated with the loss of *PARA* and *PARIE*. Taking into consideration the thermoregulatory function of the parietal organ in vertebrates [30] and that mammals and birds are endothermic, we consider that the independent loss of these genes in these lineages could be related with a change in the mechanisms regulating body temperature. As mammals and birds became less reliant on external sources of energy to maintain body temperature (evolution of endothermy [33]), the parietal organ would have degenerated (accompanied by the related signaling pathways).

### Adaptive evolution of avian opsins

Apart from the *PARA* and *PARIE* opsins, most modern birds maintained the vertebrate repertoire of opsins, suggesting that birds never became specifically adapted to limited photic conditions that might have led to the extensive pseudogenization of opsins. Additionally, since we find no global events of opsin loss during the early modern avian species radiation, birds appeared to have possessed tetrachromatic vision (*RH2*, *OPN1sw1*, *OPN1sw2* and *OPN1lw*) for most of their evolutionary history. This suggests that birds relied on a visual system specialized for discriminating different light qualities, particularly useful in complex photopic environments, where birds likely diversified.

In birds, 11 of the 15 avian opsins evolved in a non-neutral manner: *RH1*, *OPN1sw1*, *OPN1sw2*, *OPN1lw*, *TMT*, *OPN3*, *PIN*, *VA*, *RGR*, *RRH* and *OPN4x*. This suggests that visual and non-visual adaptive strategies have been imperative during avian evolution, validating the importance of the visual sense in birds. Among the visual opsins, *sw1*, *sw2* and *lw* have been more conserved in birds, while for *RH1* positive selection was found in birds and negative selection in mammals. Visual conopsins perform image-forming functions [34], and are particularly important for photopic animals, like birds. The patterns of purifying evolution in the *sw1*, *sw2* and *lw* would have permitted fine adjustment of the spectral sensitivities of these opsins, ensuring elevated photic acuity throughout avian evolution. In addition, the higher site-specific adaptive rates of *sw1* and *lw* opsins in mammals relative to birds is consistent with our preliminary analyses in Zhang *et al.* (2014) [14], where we have found that the *sw1* ω-ratio is lower in birds than in mammals (0.16/0.21 respectively). This suggests that the mechanism to maintain optimal color discrimination is more stringent in birds than in mammals.

*RH1* has the key function of conferring monochromatic vision in low light environments [35], and thus it is not surprising that it diversifies in birds that were mostly photopic-adapted. In contrast, in mammals the negative selection is consistent with their nocturnal habits and the anatomical features of the mammalian eye that are congruent with nocturnal ancestry [36]. More specifically, our findings that site 217 of *RH1* evolved under positive selection in birds is consistent with findings of positive-selection on this site in other vertebrates [26, 37]; however, it has been reported that different amino acids at these sites do not seem to cause spectral shifts in *RH1* [26]. Instead, we found an association between the T and M/A amino acid residues and the evolution of land and water neoaves clades – similar ecological condition also influenced the evolution of the olfactory receptor subgenomes [38]. Possible contributions of these specific amino acid substitutions to water and land adaptations can be tested through in-vitro experiments to verify the role of this site in *RH1* spectral tuning. Overall, the contrasting evolutionary signatures in the visual opsins between mammals and birds are consistent with their contrasting photic needs in that scotopic-adapted animals need to maximize the amount of light collected, while photopic-adapted animals require enhanced visual acuity.

### *OPN1sw1* evolution and VS/UVS vision

Our analysis suggesting that the ancestral bird possessed a VS *OPN1sw1* opsin is consistent with other analyses of amino acid variation of *sw1* spectral tuning sites that concluded that VS was the probable ancestral condition of birds [16, 39]. The ecological role of the *OPN1sw1* opsin in birds is not well understood, but is likely to be broad, as it has been associated with coloration pattern recognition [19], social signaling, hunting, nectar localization and mate-choice [6–8]. Others also suggest a role in non-visual processes such as circadian rhythm regulation [40, 41]. Species in six avian orders, Pteroclidiformes, Charadriiformes, Coraciiformes, Trogoniformes, Psittaciformes and Passeriformes, have been shown to possess UVS sensitivity [16], of which the later four belong to core landbirds. However, there are no clear patterns of coloration, breeding behaviors, activity patterns and feeding habits among the species in these groups that would explain the acquisition of UVS (or the retention of VS).

The use of UVS is most clear among Passeriforms, which is consistent with evidence of strong positive selection found in the branch leading to the passerine group for the *sw1* gene [14]. However, some Passeriformes species are also VS, which is reflected in a relative higher ω-lineage in the golden-collared manakin and American crow. These two species have been reported as cases of recent adaptation to the VS vision [42]. The contrasting root-to-tip ω-lineages appears to be an efficient methodology to study ecological adaptation scenarios in phylogenetic contexts, as it is sensitive enough to detect episodes of reversal evolution.

Another species associated with a high ω-lineage for the VS class is the paleognath tinamou. The tinamou *sw1* amino acid sequences had F, C and M residues at sites 86, 90 and 93, which corresponds with the amino acid conformation found by Ödeen *et al.* (2013) in the closely-related ostrich (*Struthio camelus*) [16]. The *sw1* sensitivity in paleognaths has been somewhat controversial, because while microspectrophotometry analysis of the *sw1* in ostrich suggests VS [18], the 86 F and 90C residues support UVS [41]. Our results, linking the tinamou with a high ω-lineage in the VS class suggests that the UVS → VS shift was relatively recent. This is congruent with the amino acid sequence similarity with UVS *sw1* and also with the microspectrophotometry analysis. Therefore, we presume that there are other amino acids in addition to 86 and 90 that likely changed the spectral sensitivity of the *sw1* photopigment, such as 93 M as suggested by Ödeen *et al.* (2013) [16].

### *MC1R*/*RH2* co-evolution

The adaptive association between the *MC1R* gene and the *RH2* visual opsin in avian species suggest that plumage colorations have been photic mediated. The *MC1R* receptor is involved in melanin-based coloration in vertebrates and is found primarily in melanocyte cells where it controls the deposition of melanin in tissues [43]. Activation of *MC1R* leads to increased synthesis of black/brown eumelanin, whereas low *MC1R* activity leads to increased synthesis of red/yellow phaeomelanin [44]. First cloned from chickens [45], several studies have found that *MC1R* is closely associated with plumage coloration [46–49]. *MC1R* adaptive evolution has also been correlated with the degree of sexual dichromatism in galliform birds, suggesting that *MC1R* may be a key link in the interaction of sexual selection and plumage colour [50]. Sexual selection would also be a reasonable explanation for the *MC1R/RH2* co-evolution. Indeed, one would expect that plumage coloration patterns would only be important for birds if they were associated with a visual system capable of “read” plumage coloration cues. Indeed, it has been shown that tetrachromatic vision, a process which requires the *RH2* photopigment, enhances plumage discriminability in birds [51]. In addition, Bloch *et al.* (2015) have suggested that rapid evolution of *RH2* in Setophaga birds (a genus of Passeriformes) is linked to sexual selection, given their exceptional plumage color diversification [52]. In situations in which sexual selection evolves in association with plumage coloration patterns and color discriminability, then there should be strong associations among *MC1R* and visual opsins. Further tests on the *sw1*, *sw2* and *lw* opsins would be welcome.

### More-recent photic adaptations in birds: the barn owl

The barn owl has very distinctive photoreceptive features relative to other birds [53, 54]. Due to a recent nocturnal adaptation, barn owls have frontally placed eyes and anatomical adaptations that improve perception of photic stimuli in low light environments; this includes an elongated eye and a high ratio between the eye and corporal sizes [53, 54]. The pseudogenization of *RH2* and the lineage-specific acceleration of *PIN* are consistent with these adaptive changes.

*RH2* is sensitive to the green photo spectrum from about 480–535 nm [2] and has undergone rapid gene loss and gain in other vertebrate lineages (reviewed in [55]). In addition, *RH2* was lost in placental mammals during the nocturnal bottleneck [3, 36]. There is evidence that some owls have a photoreceptor that is sensitive to the 503 nm spectrum, which would be consistent with a *RH2*-type photopigment [56]. If confirmed that this photopigment is from *RH2*, the pseudogenization event reported here for the barn owl would likely be lineage-specific.

*PIN* is a blue-sensitive pigment (~470 nm) expressed in the pineal gland (determined in chicken) that has a role controlling the circadian pacemaker and the rhythmic production of melatonin [57, 58]. The accelerated evolutionary rate observed in the barn owl *PIN* is appreciable (0.548/0.146, i.e. 3.7 times faster than the avian trend) and includes several non-synonymous mutations. Although it is not known if the *PIN* opsin is fully functional in the barn owl pineal gland, owls possess a rudimentary pineal with the pinealocytes having rudimentary photoreceptive features [59].The PIN protein may continue to have a role in circadian tasks associated with a nocturnal lifestyle or the degeneration of the pineal gland may have permitted the unconstrained molecular evolution of *PIN*.

Other genes that are likely to be involved in the adaptation of birds to a nocturnal life style include *sw1*, *sw2* and *lw* visual conopsins, which we were not found in the barn owl genome. Zhao *et al.* (2009a) performed phylogenetic analysis in the *lw* and *sw1* photopigments in nocturnal bats and affirmed the importance of the *sw1* in the species’ sensory ecology [60]. In particular, it would be important to determine if the *sw1* photopigment in birds is UV/UVS sensitive, as UVS vision has been associated with nocturnal habits in mammals [60, 61].

### More-recent photic adaptations in birds: the penguins

Penguins possess specialized and unique optic adaptations, including an approximately-spherical lens and a flat cornea that augment their vision when underwater [62]. At the molecular level, evidence of gene pseudogenization and positive selection in phototransduction genes have been associated with the aquatic lifestyle of the Adélie and emperor Antarctic penguins [25]. Penguin specializations include cone visual pigments tuned towards the blue-green range of the visual spectrum, presumably related with the spectral composition of their aquatic environment [63]. The retuning of the *RH2* in penguins could be linked with non-synonymous mutations in the D83, Q122, A164, A207 and S222 amino acids of *RH2* (site identification based on bovine rhodopsin homolog sites) [55, 64]. Although we did not find any variation in the two penguins for these sites, we found site deletions at S295 and K296. S295 is responsible for the red shift in *RH2* [65] while 296 K is an important retinal binding site [14]. It is not known if this causes *RH2* to be non-functional in penguins, which would require functional experimental tests. However, these indels were shared by the two penguin species, which shared a common ancestor ~23.0 mya [17], meaning that if they were deleterious (or significantly compromise the *RH2* function) we would expect the *RH2* to pseudogenize over that time period, which was not observed in any of the penguin lineages. Most likely, these indels are evidence that penguins have adapted a unique mechanism to perform the molecular interactions that mediate the photon absorption in *RH2* that is better suited for underwater environments.

Additionally, we have found evidence of accelerated evolution of *RH1*, *OPN4x* and *OPN5* in the penguin lineage. *OPN4x* is only present in non-mammalian vertebrates and is associated with non–image–forming light responses, including circadian entrainment [66–68]. The accelerated evolution of *OPN4x* suggests that penguins may have evolved new circadian responses to cope with the seasonal particularities of Antarctica, including the dramatic daily light changes and hourly differences. *OPN5* is UV-sensitive, is expressed in the chicken retina and pineal gland, and plays a role in the assistance of an 11-cis-retinal-supplying system [69]. The role of the *OPN5* in penguin vision is less obvious.

*RH1* has been associated with nocturnal/diurnal terrestrial lifestyles, but some studies have shown that *RH1* underwent shifts in spectral tuning in marine mammals [70]. Evidence from aquatic mammals are in congruence with the accelerated evolution of *RH1* in the ancestral lineage leading to penguins. Zhao *et al.* (2009b) [71] reported evidence of positive selection in the cetacean and pinniped aquatic clades, suggesting that *RH1* evolution were related with the turbid condition of aquatic environments. In addition, the occurrence of P in the 194 spectral tunning site was also verified in cetaceans, particularly in the sowerby's beaked whale (*Mesoplodon bidens*) [71]. Changes in *RH1* molecular features are compelling evidence that it contributed to penguin’s unique adaptive strategies to aquatic environments.

Similarly as for the barn owl, the emperor penguin also showed lineage-specific changes in *PIN*. However, we were unable to determine if is this is a case of adaptive or unconstrained evolution. Nevertheless, evidence suggests that the pineal organ of the Antarctic penguin (*Pygoscelis papua*) lacks typical photoreceptor elements [72], which as observed by Li *et al.* (2014), is the likely cause of the accelerated evolution/pseudogenization of the *PIN* opsin in penguins [25].

## Conclusion

The analyses of visual and non-visual opsins from 48 genomes spanning the extant avian phylogeny provide new insights on the evolutionary history of avian visual systems by revealing the molecular signatures that characterize their evolution. Our results suggest that avian adaptive strategies were driven mainly by gene loss, adaptive (negative and positive) selection and co-evolution with melanin-based plumage coloration genes. More-recent evolutionary events in the owl and penguins lineages suggest the emergence of new adaptive strategies among birds, probably promoted by the evolution of the nocturnal and aquatic lifestyles, respectively. We conclude that birds, while being remarkably dependent on the visual sense, have changed their visual and non-visual molecular systems in response to the photic environment they occupy and to the strong pressures of sexual selection.

## Methods

### Phylogenetic analysis

Psi-blast and t-blastn searches were performed employing annotated protein sequences of the zebrafinch and chicken opsin gene in the NCBI, Ensembl data bases [73, 74] and in 45 avian genomes from the Avian Phylogenomics Project [17, 75]. Additionally, plumage coloration gene sequences from the *ASIP*, *MC1R*, *TYR*, *TYRP1* and *OCA2* genes were also obtained from these databases. Accession numbers for all the sequences used in this study are compiled in Additional files 6, 11, 12 and 13.

A protein-based coding sequence alignment was performed by aligning the translated sequences using the Muscle 3.3 algorithm [76] and subsequently improved manually –gap rich and ambiguous regions were removed. Partial sequences were excluded from phylogenetic analysis. The presence of saturation in base substitution for each of the alignments was tested using DAMBE5 [20]. None of the alignments showed evidence of saturation bias (Additional file 14) and thus, all were acceptable for phylogenetic analysis. jModelTest (version 0.1.1) with Akaike Information criterion (AIC) was used to estimate the most appropriate nucleotide substitution model [77]. For all genes the GTR + I + Γ model was selected as the most-appropriate model with 95 % confidence.

### Site- and branch-specific selection analyses

Opsin gene alignments and the species tree were used in the codeml package in PAML 4.4 software [78] to assess the site- and branch-specific codon substitution models of maximum likelihood. The genome-scale avian species tree [17] was used to perform the site and branch-specific selection analysis because we aim to trace gene evolution within a framework of species evolution and to eliminate possible confounding effects of gene tree phylogeny variation or error. The site specific models were tested comparatively: M7 (beta) *vs* M8 (beta + ω) and M8a (beta + ω = 1) *vs* M8 [22]. Subsequently, likelihood rate comparisons (LRT) were performed to test which models best fit the data. M7 and M8 assume a β-distribution for the ω value between 0 and 1 but M8 additionally allows the occurrence of positively-selected sites (ω > 1). M8a tests for neutral evolution by fixing the parameter ω = 1. Whenever the likelihood ratio test was significant under the M8 model, the Bayes Empirical Bayes (BEB) method was used to calculate posterior probabilities of the sites that are subject to positive selection (accepted at > 0.95) [79]. Site-selection analysis were implemented in PAML4 package [78].

The branch selection models were implemented comparing the estimated ω-ratio for all lineages in the tree (one-ratio model) and the two-ratios model, which assigns two ω ratios for the foreground and background branches [80]. The foreground ω-lineage maximum likelihood estimation was calculated for each species of each gene labeling the tip, the root and all intermediate branches. For the species-specific branch analysis on the barn owl terminal lineage and the emerging lineage of penguins, we test the significance of the ω variations (one-ratio *vs.* two-ratio model with 1 degree of freedom [80]) using the LRT to perform the hypothesis testing.

### Statistical analysis

We implemented a phylogenetic logistic regression that explains the VS or UVS *sw1* sensitivity in each species using the ω-lineage using phylolm package in R software [81]. Additionally, a robust principal component analysis of ω-lineage values was conducted following the Hubert (2005) approach [82], in order to assess the avian opsin outlier map, which summarizes those lineages that show unexpected ω-tendencies in the evolutionary dimensional space of the studied opsins.

To seek evidence for opsin and plumage coloration gene co-evolution we used the ω-lineage foreground rate of evolution, classified in three evolutionary categories: accelerated (A, more than 0.75 quartile), conserved (C, less than the 0.25 quartile) or neutral (N, otherwise). Association tests for each visual-coloration gene pairs were implemented considering those lineages that showed the same evolutionary behavior: AA, CC or NN. Since we were interested in these particular 3 coevolving behaviors (amongst nine possibilities), we implemented a proportion test, assuming that the null hypothesis *p* = 6/16. Under independence, 6/16 is the expected proportion of lineages showing the same evolutionary behavior. Associations with less than 15 comparative pairs were excluded to avoid false positives and the Bonferroni correction was applied for multiple association tests (59 multiple tests, *p*-value = 8.4 x 10^−4^). All statistical analyses were performed using the R software [81].

### Availability of supporting data

The data sets supporting the results of this article are included within the article and its additional files.

## Additional files

Additional file 1:
**GC ratio comparisons for avian opsins. **The GC ratio corresponds to the %GC / [%G * %C] ratio. GC ratio equal to 1 corresponds to the absence of GC bias, while value lees than 0.76 or higher than 1.25 (horizontal red lines in the plot) indicate deviances in GC use. (PDF 12 kb) Additional file 2:
**Log GC ratio pairwise comparisons between the avian opsins.** The presented *p*-values are corrected using the Bonferroni adjustment. *p*-values colored in red represent statistically non-significant differences between the average log GC ratios of the compared genes. Pairwise comparisons for the *sw1*, *sw2* and *lw* opsins are highlighted in grey. (PDF 27 kb) Additional file 3:
**Site-selection tests for the avian and mammalian opsins and the melanin-based coloration genes.** The logarithm of the model likelihood is represented by lnL, the number of model parameters are represented by np and the LRT is the likelihood ratio test. Accepted site-selection model are indicated with an asterisk (*) when the M7 model of negative selection is statistically significant, or a double asterisk (**) if the M8 model of positive selection is statically significant. All the LRT comparisons were performed assuming a significance level of 0.05. (PDF 103 kb) Additional file 4:
**Phylogenetic reconstruction of the** ***RH1*** **217 residue in birds.** The species tree was used as described by Jarvis *et al.* (2014) [17]. Red and blue species represent land and water birds respectively. Colored ancestral lineages are suggestive of the M (Met; red), L (Leu; blue) and A (Ala; violet) residues in the 217 site of the *RH1* opsin. (PDF 19 kb) Additional file 5:
**Species-specific ω-lineage estimates.** The ω-lineage estimates under the assumption of the branch-specific two-ratios model. Species-specific ω-lineage was calculated considering the species tree and the root-to-tip labeling. (PDF 67 kb) Additional file 6:
**UVS/VS condition for avian** ***sw1*** **sequences.** Avian *sw1* sequences used in this study and the respective accession numbers. UVS/UV *sw1* type was inferred using the 86, 90 and 93 spectral tuning sites (highlighted in red). Amino acid combinations were compared with previously published sequences in Ödeen *et al.* (2013) [16]. (PDF 174 kb) Additional file 7:
**Amino acid sequence of the barn owl (** ***Tyto alba*** **) RH2 opsin.** *RH2* conopsin multiple sequence alignment of the barn owl and the zebra finch (Neoaves representative), chicken (Galloanseres representative) and ostrich (Paleognathae representative). Regions in red indicate special features of the barn owl *RH2* sequence: non-synonymous mutations, indels and stop-codons. (PDF 130 kb) Additional file 8:
**Species-specific branch selection tests.** The one-ratio model (H0) was tested against the two-ratios model considering the alternative hypotheses of verifying differentiated ω-ratios in the terminal lineage of the barn owl (H1.1) or the emerging lineage of penguins (H1.2). Significant alternative hypothesis are marked with a red *p*-value. lnL is the logarithm of the model likelihood and the LRT is the likelihood ratio test. All the LRT comparisons were performed with 1 degree of freedom and assuming a significance level of 0.05. (PDF 147 kb) Additional file 9:
**Co-evolution between opsins and the melanin-based plumage coloration genes in birds.** Association tests were implemented using the ω-lineage classified in three categories: accelerated, conserved and neutral. The association between two genes was measured using the proportion of lineages showing the same evolutionary behavior and considering the alternative hypothesis *p* > 6/16. Values in the table correspond to the Pearson's χ^2^ test statistic and the sample proportions (in brackets). Significant (*p*-value < 8.4 x 10^−4^) and strong (*p*-value < 0.05) associations are indicated in red-bold and bold, respectively. (PDF 92 kb) Additional file 10:
**Genomic localization of the genes used for the co-evolution analysis.** Genomic localization of the visual genes (in orange) and melanin-based plumage coloration genes (in red) in the chicken and zebra finch karyotypes. (PDF 15 kb) Additional file 11:
**Avian opsin and melanin-based plumage coloration sequences.** Accession numbers are indicated for the complete sequences, while for partial sequences the genomic location is given. Asterisk (*) indicate that we were not able to identify the gene in the respective genome. Gene sequences are available in the Avian Phylogenomics Project database (http://avian.genomics.cn/en/, [75]). (PDF 205 kb) Additional file 12:
**Avian** ***sw2*** **and** ***lw*** **sequences.** Accession number of the *sw2* and *lw* sequences used for site selection analyses. (PDF 12 kb) Additional file 13:
**Mammalian opsin sequences.** Accession number of the mammalian opsins sequences used for site selection analyses. (PDF 88 kb) Additional file 14:
**Saturation analysis.** The presence of saturation in base substitution for each of the gene alignment was tested by comparing half of the theoretical saturation index expected when assuming full saturation (Iss.c, critical value) with the observed saturation index (Iss). Absence of substitution saturation is verified when Iss is lower than Iss.c for a significant *p*-value. (PDF 24 kb)

## Abbreviations

AIC: Akaike Information criterion
GC: Guanine-cytosine dinucleotide
LRT: Likelihood ratio test
UVS: Ultra-violet sensitivity
VS: Violet sensitivity
