# Supplementary figures and images for: Gene loss, adaptive evolution and the co-evolution of plumage coloration genes with opsins in birds
Source: BMC Genomics. 2015 Oct 6;16:751. doi: 10.1186/s12864-015-1924-3 (PMC4595237; doi:10.1186/s12864-015-1924-3)

%CG  
%C %G

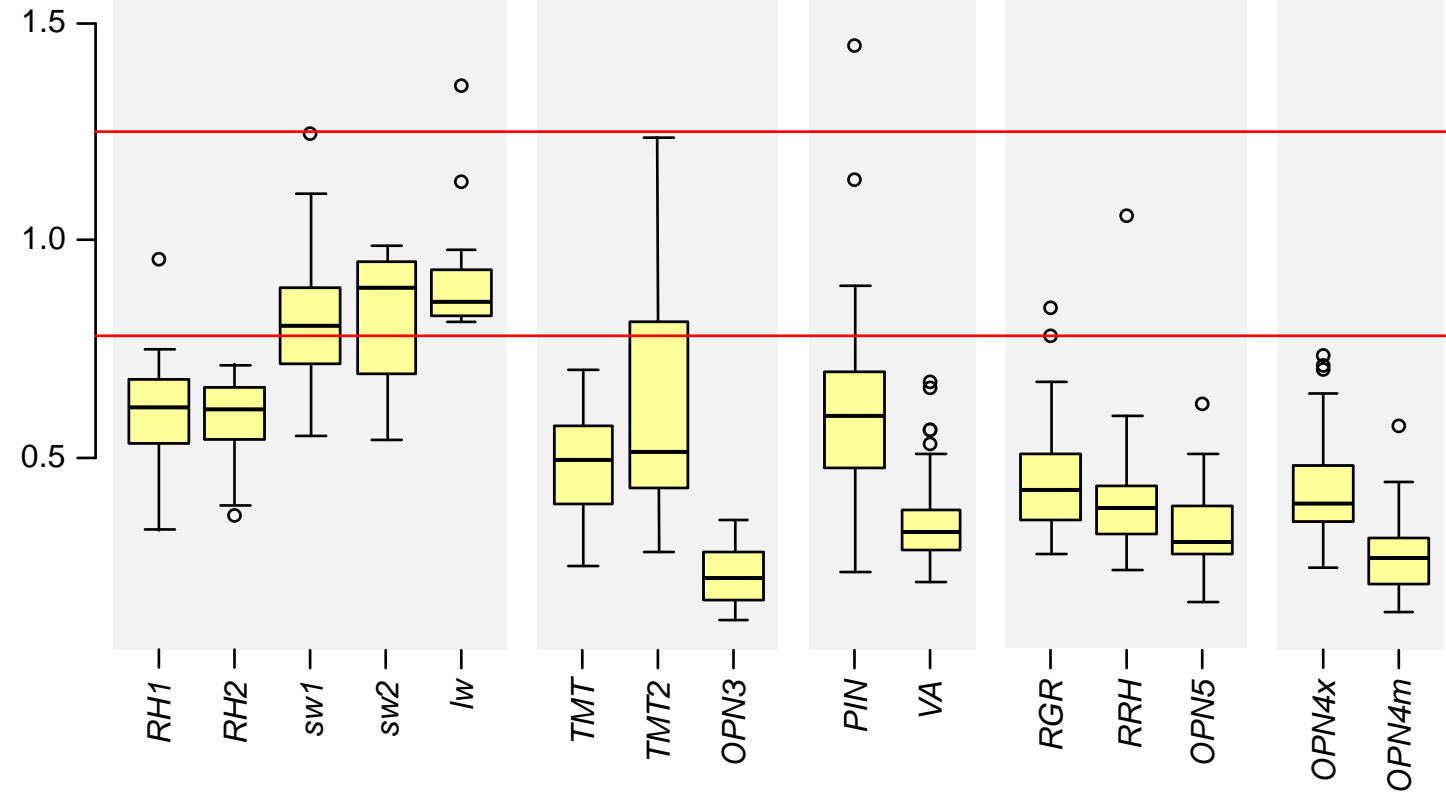

Supplement: Additional file 1: — GC ratio comparisons for avian opsins. The GC ratio corresponds to the %GC / [%G * %C] ratio. GC ratio equal to 1 corresponds to the absence of GC bias, while value lees than 0.76 or higher than 1.25 (horizontal red lines in the plot) indicate deviances in GC use. (PDF 12 kb) [file 12864_2015_1924_MOESM1_ESM.pdf]

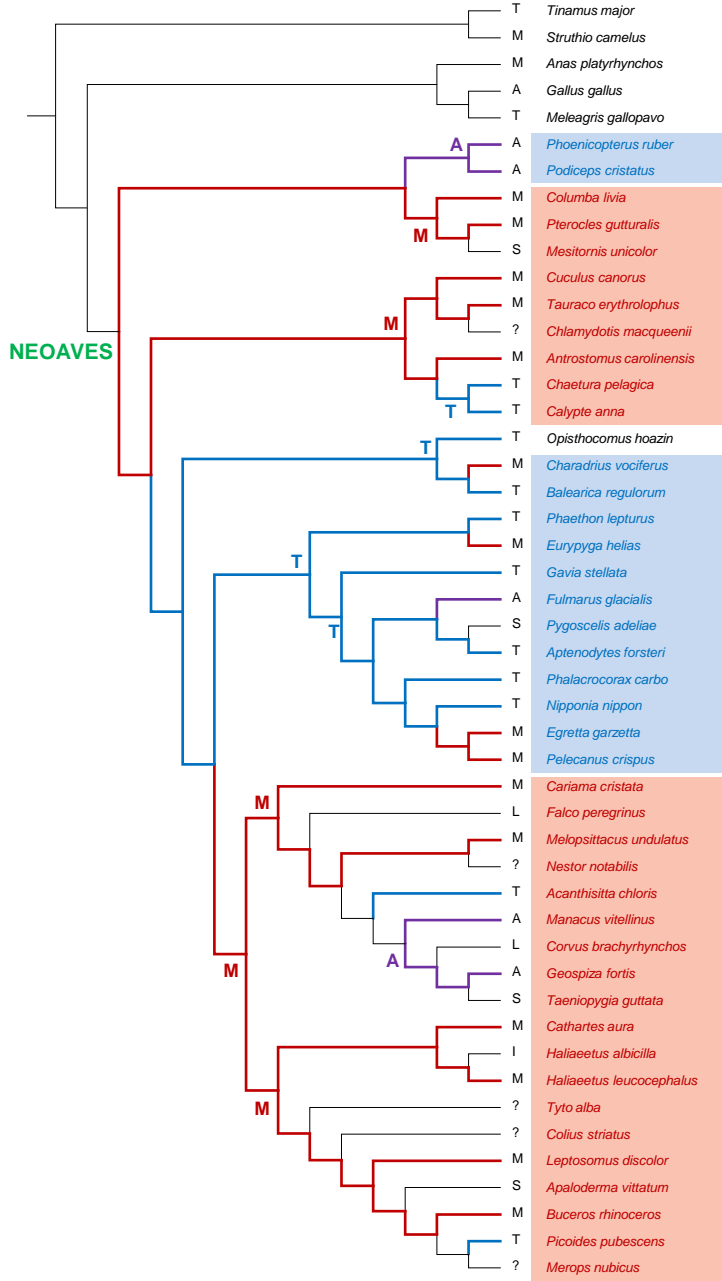

Supplement: Additional file 4: — Phylogenetic reconstruction of the RH1 217 residue in birds. The species tree was used as described by Jarvis et al. (2014) [17]. Red and blue species represent land and water birds respectively. Colored ancestral lineages are suggestive of the M (Met; red), L (Leu; blue) and A (Ala; violet) residues in the 217 site of the RH1 opsin. (PDF 19 kb) [file 12864_2015_1924_MOESM4_ESM.pdf]

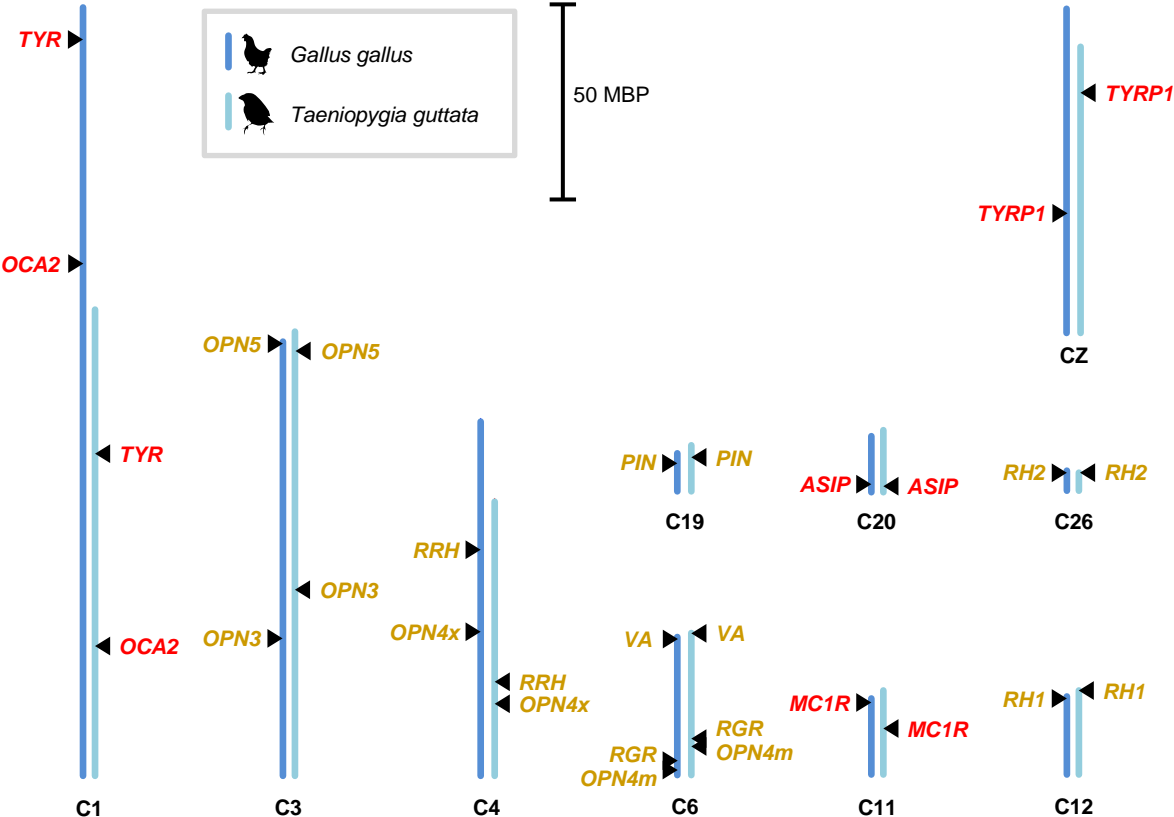

Supplement: Additional file 10: — Genomic localization of the genes used for the co-evolution analysis. Genomic localization of the visual genes (in orange) and melanin-based plumage coloration genes (in red) in the chicken and zebra finch karyotypes. (PDF 15 kb) [file 12864_2015_1924_MOESM10_ESM.pdf]
