# Supplementary material for: Gene loss, adaptive evolution and the co-evolution of plumage coloration genes with opsins in birds
Source: BMC Genomics. 2015 Oct 6;16:751. doi: 10.1186/s12864-015-1924-3 (PMC4595237; doi:10.1186/s12864-015-1924-3)
Supplement: Additional file 2: — Log GC ratio pairwise comparisons between the avian opsins. The presented p-values are corrected using the Bonferroni adjustment. p-values colored in red represent statistically non-significant differences between the average log GC ratios of the compared genes. Pairwise comparisons for the sw1, sw2 and lw opsins are highlighted in grey. (PDF 27 kb) [file 12864_2015_1924_MOESM2_ESM.pdf]

	lw	*OPN3*	*OPN4m*	*OPN4x*	*OPN5*	*PIN*	*RGR*	*RH1*	*RH2*	*RRH*	*sw1*	*sw2*	*TMT*	*TMT2*
OPN3	0.000	.												
OPN4m	0.000	0.081	.											
OPN4x	0.000	0.000	0.000	.										
OPN5	0.000	0.000	0.002	0.000	.									
PIN	0.000	0.000	0.000	0.000	0.000	.								
RGR	0.000	0.000	0.000	1.000	0.000	0.000	.							
RH1	0.000	0.000	0.000	0.000	0.000	1.000	0.000	.						
RH2	0.000	0.000	0.000	0.000	0.000	1.000	0.000	1.000	.					
RRH	0.000	0.000	0.000	1.000	0.112	0.000	0.278	0.000	0.000	.				
sw1	1.000	0.000	0.000	0.000	0.000	0.000	0.000	0.000	0.000	0.000	.			
sw2	1.000	0.000	0.000	0.000	0.000	0.003	0.000	0.006	0.002	0.000	1.000	.		
TMT	0.000	0.000	0.000	1.000	0.000	0.195	1.000	0.108	0.195	0.108	0.000	0.000	.	
TMT2	0.000	0.000	0.000	0.000	0.000	1.000	0.000	1.000	1.000	0.000	0.000	0.002	0.173	.
VA	0.000	0.000	0.000	0.026	1.000	0.000	0.002	0.000	0.000	1.000	0.000	0.000	0.002	0.000
