# Supplementary material for: Gene loss, adaptive evolution and the co-evolution of plumage coloration genes with opsins in birds
Source: BMC Genomics. 2015 Oct 6;16:751. doi: 10.1186/s12864-015-1924-3 (PMC4595237; doi:10.1186/s12864-015-1924-3)
Supplement: Additional file 3: — Site-selection tests for the avian and mammalian opsins and the melanin-based coloration genes. The logarithm of the model likelihood is represented by lnL, the number of model parameters are represented by np and the LRT is the likelihood ratio test. Accepted site-selection model are indicated with an asterisk (*) when the M7 model of negative selection is statistically significant, or a double asterisk (**) if the M8 model of positive selection is statically significant. All the LRT comparisons were performed assuming a significance level of 0.05. (PDF 103 kb) [file 12864_2015_1924_MOESM3_ESM.pdf]

A. Site-selection for avian opsins

Gene	lnL M7	np	lnL M8	np	LRT 7-8	*p*-value	lnL M8a	np	LRT 8a-8	*p*-value	ω	
RH1	-7281.893	95	-7259.274	97	45.238	0.000	-7269.845	96	21.142	0.000	0.061	**
RH2	-8492.132	93	-8473.652	95	36.959	0.000	-8474.673	94	2.041	0.153	0.080	
OPN1sw1	-5772.490	65	-5769.586	67	5.808	0.055	-5769.697	66	0.223	0.637	0.041	*
OPN1sw2	-3695.364	27	-3695.367	29	0.000	1.000	-3695.594	28	0.453	0.501	0.050	*
OPN1lw	-2472.949	33	-2472.952	35	0.000	1.000	-2472.648	34	0.000	1.000	0.024	*
TMT	-2850.553	35	-2850.394	37	0.320	0.852	-2850.392	36	0.000	1.000	0.220	*
TMT2	-4682.709	97	-4678.257	99	8.904	0.012	-4678.322	98	0.130	0.719	0.118	
OPN3	-3619.953	89	-3617.762	91	4.381	0.112	-3617.821	90	0.119	0.730	0.101	*
PIN	-12231.617	81	-12191.501	83	80.231	0.000	-12202.455	82	21.908	0.000	0.230	**
VA	-8654.677	91	-8634.321	93	40.713	0.000	-8641.777	92	14.912	0.000	0.265	**
RGR	-7439.267	97	-7421.642	99	35.251	0.000	-7434.286	98	25.288	0.000	0.148	**
RRH	-6693.016	97	-6673.678	99	38.677	0.000	-6680.310	98	13.264	0.000	0.155	**
OPN5	-8232.778	93	-8225.471	95	14.616	0.001	-8226.468	94	1.995	0.158	0.112	
OPN4x	-11033.939	97	-11012.902	99	42.074	0.000	-11022.799	98	19.794	0.000	0.171	**
OPN4m	-7206.853	93	-7200.398	95	12.911	0.002	-7201.795	94	2.796	0.095	0.203	

B. Site-selection for avian melanin-based plumage coloration genes

Gene	lnL M7	np	lnL M8	np	LRT 7-8	*p*-value	lnL M8a	np	LRT 8a-8	*p*-value	ω	
MC1R	-4933.893	45	-4932.207	47	3.371	0.185	-4931.955	46	0.000	1.000	0.065	*
TYR	-14405.107	97	-14378.035	99	54.143	0.000	-14384.898	98	13.725	0.000	0.209	**
TYRP1	-9591.835	83	-9578.852	85	25.966	0.000	-9579.190	84	0.677	0.411	0.078	
OCA2	-15119.542	79	-15094.068	81	50.947	0.000	-15110.967	80	33.797	0.000	0.196	**
ASIP	-5477.254	83	-5447.139	85	60.231	0.000	-5459.776	84	25.275	0.000	0.429	**

C. Site-selection for mammalian opsins

Gene	lnL M7	np	lnL M8	np	LRT 7-8	*p*-value	lnL M8a	np	LRT 8a-8	*p*-value	ω	
RH1	-18060.196	251	-18060.200	253	0.000	1.000	-18056.699	252	0.000	1.000	0.064	*
OPN1sw1	-17626.250	199	-17605.265	201	41.971	0.000	-17607.898	200	5.266	0.022	0.178	**
OPN1lw	-20776.122	253	-20769.910	255	12.424	0.002	-20766.851	254	0.000	1.000	0.135	
OPN3	-14209.085	127	-14171.960	129	74.251	0.000	-14185.161	128	26.402	0.000	0.310	**
RGR	-15640.311	155	-15634.514	157	11.596	0.003	-15633.702	156	0.000	1.000	0.193	
RRH	-16299.456	165	-16286.261	167	26.391	0.000	-16295.620	166	18.717	0.000	0.273	**
OPN5	-11367.748	163	-11360.527	165	14.441	0.001	-11361.359	164	1.664	0.197	0.089	
OPN4m	-26002.747	169	-25972.711	171	60.072	0.000	-25981.877	170	18.333	0.000	0.239	**
