# Supplementary material for: Gene loss, adaptive evolution and the co-evolution of plumage coloration genes with opsins in birds
Source: BMC Genomics. 2015 Oct 6;16:751. doi: 10.1186/s12864-015-1924-3 (PMC4595237; doi:10.1186/s12864-015-1924-3)
Supplement: Additional file 5: — Species-specific ω-lineage estimates. The ω-lineage estimates under the assumption of the branch-specific two-ratios model. Species-specific ω-lineage was calculated considering the species tree and the root-to-tip labeling. (PDF 67 kb) [file 12864_2015_1924_MOESM5_ESM.pdf]

Species	*RH1*	*RH2*	*sw1*	*OPN4m*	*OPN4x*	*OPN3*	*TMT*	*TMT2*	*PIN*	*VA*	*RGR*	*RRH*	*OPN5*	*MC1R*	*TYR*	*TYRP1*	*OCA2*	*ASIP*
Acanthisitta chloris	0.076	0.031		0.219	0.163	0.026		0.133		0.684	0.235	0.169	0.128	0.067	0.216	0.06	0.097	
Anas platyrhynchos	0.007	0.013		0.087	0.079	0.09		0.04	0.074	0.125	0.068	0.099	0.082	0.026	0.156		0.113	0.648
Apaloderma vittatum	0.021	0.057		0.109	0.104	0.053		0.045	0.116	0.182	0.114	0.21	0.058		0.141	0.073		0.35
Aptenodytes forsteri	0.087	0.092		0.219	0.193	0.204	0.306	0.102	0.38	0.18	0.105	0.203	0.149	0.101	0.088	0.069	0.098	0.204
Balearica regulorum	0.026			0.171	0.078	0.082		0.12	0.077	0.151	0.075	0.171	0.027		0.113		0.141	0.368
Buceros rhinoceros	0.018	0.018		0.217	0.115	0.167		0.105	0.068	0.302	0.089	0.172	0.044		0.099	0.062	0.105	
Calypte anna	0.025	0.017		0.171	0.199	0.113		0.1	0.109	0.265	0.134	0.158	0.082		0.293	0.155	0.338	0.15
Caprimugus carolinensis	0.069	0.041		0.113	0.15	0.161	0.284	0.089	0.078	0.134	0.124	0.269	0.06		0.175	0.088	0.132	0.125
Cariama cristata	0	0.015		0.217	0.095	0.687		0.074	0.11	0.338	0.164	0.368	0.029		0.085	0.063	0.135	0.38
Cathartes aura	0.018	0.065		0.226	0.113	0.098		0.107		0.301	0.086	0.186		0.146	0.159	0.061	0.134	0.505
Chaetura pelagica	0.023	0.021		0.229	0.172	0.086		0.097	0.126	0.16	0.222	0.167	0.043	0.034	0.283	0.157	0.207	
Charadrius vociferus	0.021	0.011		0.109	0.083	0.132	0.275	0.078	0.067	0.149	0.097	0.156	0.051	0.029	0.14	0.049	0.216	0.191
Chlamydotis macqueenii	0.017	0.032		0.071	0.149	0.055		0.162	0.079	0.139	0.14	0.197	0.064		0.173	0.069	0.284	0.214
Colius striatus		0		0.13	0.1	0.017		0.073	0.103	0.169	0.08	0.173	0.07		0.163	0.072		0.263
Columba livia	0.014	0.012	0.039	0.173	0.089	0.111		0.304	0.068	0.151	0.093	0.201	0.087	0.073	0.175	0.095	0.164	0.12
Corvus brachyrhynchos	0.036	0.052	0.064	0.377	0.098	0.071	0.143	0.108	0.103	0.493	0.18	0.246	0.092	0.071	0.244	0.066	0.13	0.241
Cuculus canorus	0.026	0.017	0.005	0.16	0.149	0.042		0.108	0.111	0.16	0.107	0.185	0.079	0.035	0.138	0.047	0.135	0.203
Egretta garzetta	0.018	0.016	0.041		0.082		0.061	0.054	0.087	0.157	0.068	0.186	0.143		0.171		0.14	0.393
Eurypyga helias	0.069	0.022		0.086	0.168	0.075	0.115	0.069	0.067	0.128	0.158	0.151	0.081		0.128	0.065	0.128	0.171
Falco peregrinus	0.017	0.011	0.018	0.217	0.15	0.131		0.065		0.196	0.16	0.196	0.039	0.033	0.119	0.05	0.124	0.32
Fulmarus glacialis	0.026	0.024		0.228	0.134	0.082	0.305	0.095		0.161	0.062	0.294	0.109		0.192	0.158		0.451
Gallus gallus	0.016	0.006	0.018	0.139	0.112	0.064		0.162	0.08	0.168	0.089	0.066	0.115	0.016	0.2	0.063	0.131	0.218
Gavia stellata	0.027	0.031		0.238	0.223	0.048	0.319	0.143		0.17	0.167	0.169	0.059		0.095	0.036	0.143	0.276
Geospiza fortis	0.018	0.031	0.06	0.245	0.115	0.109	0.245	0.084	0.098	0.521	0.146	0.135	0.086	0.042	0.197	0.054	0.122	0.238
Haliaeetus albicilla	0.065	0.015		0.078	0.134	0.203		0.143	0.1	0.279	0.09	0.173	0.018		0.166	0.061		0.566
Haliaeetus leucocephalus	0.032	0.014	0.011	0.078	0.14	0.243		0.143	0.054	0.177	0.09	0.173	0.017	0.084	0.166	0.061	0.118	0.281
Leptosomus discolor	0.023	0.013		0.112	0.096		0.267	0.093	0.096	0.178	0.109	0.128	0.039		0.227	0.054	0.16	
Manacus vitellinus	0.022	0.092	0.068	0.249	0.142	0.081		0.115	0.092	0.353	0.288	0.204	0.122	0.047	0.178	0.076	0.094	0.233
Meleagris gallopavo	0.008	0.008		0.129	0.084	0.039		0.167	0.093	0.167	0.075	0.071	0.111	0.008	0.163		0.13	0.169
Melopsittacus undulatus	0.011	0.025	0.048	0.239	0.13	0.069		0.087	0.09	0.405	0.11	0.115	0.042	0.045	0.107	0.091	0.112	0.542
Merops nubicus	0.019	0.015		0.224	0.112	0.041		0.081	0.083	0.19	0.081	0.167	0.057	0.063	0.102	0.09	0.117	
Mesitornis unicolor	0.023	0.022		0.23	0.098	0.244	0.281	0.131	0.11	0.119	0.103	0.332	0.082	0.04	0.202	0.138	0.144	0.17
Nestor notabilis	0.015	0.014	0.051	0.273	0.142	0.151		0.064	0.13	0.395	0.114	0.184	0.05	0.044	0.118	0.077		0.561
Nipponia nippon	0.012	0.022	0.042	0.249	0.146	0.075	0.311	0.057	0.108	0.149	0.071	0.272	0.04	0.138	0.149	0.077	0.167	
Opisthocomus hoazin	0.016	0.02		0.1	0.121	0.204		0.13	0.089	0.154	0.185	0.384	0.096		0.103	0.06	0.164	0.211
Pelecanus crispus	0.015	0.021		0.236	0.063	0.114	0.27	0.103	0.09	0.126	0.09	0.341	0.033		0.1			0.463
Phaethon lepturus	0.029	0.09		0.226	0.162	0.048		0.083	0.064	0.131	0.114	0.327	0.051		0.076	0.079	0.134	0.13
Phalacrocorax carbo	0.077	0.033	0.058	0.243	0.096	0.27		0.104	0.102	0.135	0.093	0.203	0.067		0.112	0.035		0.441
Phoenicopterus ruber	0.015	0.031		0.221	0.122	0.063		0.042	0.056	0.137	0.076	0.245			0.074	0.109		0.145
Picoides pubescens	0.027	0.011	0.045	0.226	0.106	0.139	0.27	0.137	0.084	0.291	0.089	0.171	0.082	0.041	0.094	0.09	0.149	0.216
Podiceps cristatus	0.025	0		0.135	0.088	0.108	0.315	0.032	0.082		0.096	0.161	0.029		0.092	0.066	0.131	
Pterocles gutturalis	0.069	0.012		0.217	0.115			0.18	0.075	0.159	0.095	0.164	0.042		0.318	0.067	0.128	0.285
Pygoscelis adeliae	0.079	0.208		0.232	0.225	0.095	0.343	0.123		0.172	0.105	0.22	0.185		0.135	0.137	0.098	0.322
Struthio camelus	0.022	0.012			0.079			0.113		0	0.105	0.084	0.142		0.107		0.127	0.838
Taeniopygia guttata	0.019	0.031	0.065	0.247	0.13	0.103		0.122	0.103	0.508	0.186	0.145	0.081	0.062	0.2	0.075	0.132	0.172
Tauraco erythrolophus	0.024	0.017		0.245	0.097	0.043		0.093	0.081	0.174	0.087	0.199	0.092		0.092	0.056	0.19	0.134
Tinamus guttatus	0.01	0.014	0.074		0.117			0.117			0.047	0.116	0.094		0.173	0	0.13	0.217
Tyto alba	0.029			0.247	0.128	0.054		0.101	0.402		0.157	0.253	0.051		0.131			0.598
