# Supplementary material for: Gene loss, adaptive evolution and the co-evolution of plumage coloration genes with opsins in birds
Source: BMC Genomics. 2015 Oct 6;16:751. doi: 10.1186/s12864-015-1924-3 (PMC4595237; doi:10.1186/s12864-015-1924-3)
Supplement: Additional file 6: — UVS/VS condition for avian sw1 sequences. Avian sw1 sequences used in this study and the respective accession numbers. UVS/UV sw1 type was inferred using the 86, 90 and 93 spectral tuning sites (highlighted in red). Amino acid combinations were compared with previously published sequences in Ödeen et al. (2013) [16]. (PDF 174 kb) [file 12864_2015_1924_MOESM6_ESM.pdf]

Order	Species	Assession number	ω backgroud	ω foreground	ratio	aa seq 84-94	Type
Accipitriformes	*Haliaeetus leucocephalus*	XM_010568780.1	0.038	0.011	0.289	L M C C V F C I F T V	VS
Columbiformes	*Columba livia*	NM_001282821.1	0.035	0.039	1.114	F L A C I I C I F T V	VS
Cuculiformes	*Cuculus canorus*	XM_009563301.1	0.039	0.005	0.128	F I C C L F S V F T V	VS
Falconiformes	*Falco cherrug*	XM_005446545.1	0.038	0.018	0.474	L M C C V F C I F T V	VS
Galliformes	*Gallus gallus*	NM_205438.1	0.039	0.018	0.462	F I S C I F S V F T V	VS
Passeriformes	*Corvus brachyrhynchos*	XM_008637700.1	0.032	0.064	2.000	F L A C I F C I F T V	VS
Passeriformes	*Euplectes afer*	AY274223.1	0.031	0.072	2.323	F L A C I F C I F T V	UVS
Passeriformes	*Euplectes orix*	AY274224.1	0.031	0.07	2.258	F L A C I F C I F T V	UVS
Passeriformes	*Ficedula albicollis*	XM_005061513.1	0.031	0.063	2.032	L M C C V F C I F T V	UVS
Passeriformes	*Geospiza fortis*	XM_005430818.1	0.032	0.06	1.875	L M C C V F C I F T V	UVS
Passeriformes	*Luscinia calliope*	AY274226.1	0.031	0.061	1.968	L M C C V F C I F T V	UVS
Passeriformes	*Luscinia svecica*	AY274225.1	0.031	0.059	1.903	L M C C V F C I F T V	UVS
Passeriformes	*Manacus vitellinus*	XM_008925921.1	0.032	0.068	2.125	L M C C V F C I F T V	VS
Passeriformes	*Parus caeruleus*	AY274220.1	0.032	0.057	1.781	F I S C I F S V F T V	UVS
Passeriformes	*Pseudopodoces humilis*	XM_005533413.1	0.032	0.059	1.844	F I S C I F S V F T V	UVS
Passeriformes	*Parus major*	AY274221.1	0.032	0.062	1.938	L M C C V F C I F T V	UVS
Passeriformes	*Parus palustris*	AY274222.1	0.031	0.068	2.194	F I S C I F S V F T V	UVS
Passeriformes	*Serinus canaria*	NM_001302100.1	0.03	0.071	2.367	F L A C I F C I F T V	UVS
Passeriformes	*Taeniopygia guttata*	NM_001076704.1	0.031	0.065	2.097	F L S C I F S V F T V	UVS
Pelecaniformes	*Egretta garzetta*	XM_009645256.1	0.035	0.041	1.171	L M C C V F C I F T V	VS
Pelecaniformes	*Nipponia nippon*	XM_009468025.1	0.035	0.042	1.200	L M C C V F C I F T V	VS
Pelecaniformes	*Phalacrocorax carbo*	EF568933.1	0.034	0.058	1.706	F L C C I F S V F T V	VS
Piciformes	*Picoides pubescens*	HM150794.1	0.035	0.045	1.286	F M C C I F S V F T V	VS
Psittaciformes	*Barnardius zonarius*	HM150799.1	0.033	0.052	1.576	F V S C V L S V F V V	UVS
Psittaciformes	*Cacatua galerita*	HM150802.1	0.034	0.045	1.324	F L A C I F C I F T V	UVS
Psittaciformes	*Calyptorhynchus latirostris*	HM150800.1	0.034	0.045	1.324	F I S C I F S V F T V	UVS
Psittaciformes	*Eolophus roseicapillu*	HM150801.1	0.034	0.05	1.471	F L A C I F C I F T V	UVS
Psittaciformes	*Melopsittacus undulatus*	Y11787.1	0.034	0.048	1.412	L M C C V F C I F T V	UVS
Psittaciformes	*Nestor notabilis*	XM_010021998.1	0.034	0.051	1.500	L M C C V F C I F T V	UVS
Psittaciformes	*Platycercus elegans*	KF134492.1	0.034	0.051	1.500	F I S C I F S V F T V	UVS
Sphenisciformes	*Spheniscus humboldti*	AJ277991.1	0.036	0.031	0.861	F I S C I F S V F T V	VS
Tinamiformes	*Tinamus guttatus*	XM_010211816.1	0.035	0.074	2.114	F I F C V F C V F M V	VS
