# Supplementary material for: Gene loss, adaptive evolution and the co-evolution of plumage coloration genes with opsins in birds
Source: BMC Genomics. 2015 Oct 6;16:751. doi: 10.1186/s12864-015-1924-3 (PMC4595237; doi:10.1186/s12864-015-1924-3)
Supplement: Additional file 7: — Amino acid sequence of the barn owl ( Tyto alba ) RH2 opsin. RH2 conopsin multiple sequence alignment of the barn owl and the zebra finch (Neoaves representative), chicken (Galloanseres representative) and ostrich (Paleognathae representative). Regions in red indicate special features of the barn owl RH2 sequence: non-synonymous mutations, indels and stop-codons. (PDF 130 kb) [file 12864_2015_1924_MOESM7_ESM.pdf]

1. Barn owl
2. Zebra finch
3. Chicken
4. Ostrinch

```
   MNGT-GVNFY MPMSNKTGLV R--------- -AEPWKYHVV CCYIFFLIST GSPINLLTLL  60
   MNGTEGINFY VPMSNKTGVV RSPFEYPQYY LAEPWKYRLV CCYIFFLIST GFPINFLTLL
   MNGTEGINFY VPMSNKTGVV RSPFEYPQYY LAEPWKYRLV CCYIFFLIST GLPINLLTLL
   MNGTEGINFY VPMSNKTGVV RSPFEYPQYY LAEPWKYRVV CCYIFFLIST GLPINLLTLL

1  VTFKHKKLRQ PLNYILVNLA VADLFMACFG FTVTFYSAWN GSFVFGPVGC AVEGFFATLG  120
2  VTFKHKKLRQ PLNYILVNLA VADLCMACFG FTVTFYTAWN GYFVFGPIGC AVEGFFATLG
3  VTFKHKKLRQ PLNYILVNLA VADLFMACFG FTVTFYTAWN GYFVFGPVGC AVEGFFATLG
4  VTFKHKKLRQ PLNYILVNLA VADLFMACFG FTVTFYTAWN GYFVFGPVGC AVEGFFATLG

1  GQVALWSLVV LAIEHYIIIC KPMGNFRFST THAMGGIAFT WIMALSCAAP PLFG*SRYMP  180
2  GQVALWSLVV LAIERYIVIC KPMGNFRFSA SHALMGIAFT WVMAISCAAP PLFGWSRYIP
3  GQVALWSLVV LAIERYIVVC KPMGNFRFSA THAMMGIAFT WVMAFSCAAP PLFGWSRYMP
4  GQVALWSLVV LAIERYIVVC KPMGNFRFSS SHAMMGIAFT WVMAFSCAAP PLFGWSRYMP

1  EGMQCSCSPD YCTHNADYYN ESYILCMFII HFIIHVMVIF FSCRRLICKV RGNAGKRHGP  240
2  EGMQCSCGPD YYTHNPDFHN ESYVLYMFVI HFIIPVVIIF FSYGRLVCKV REAAAQQQES
3  EGMQCSCGPD YYTHNPDYHN ESYVLYMFVI HFIIPVVVIF FSYGRLICKV REAAAQQQES
4  EGMQCSCGPD YYTHNPDYHN ESYVLYMFVI HFIIPVVVIF FSYGRLICKV REAAAQQQES

1  GWAGEG---- TCMVILMVLG FMLSWTPYAV VAFWIFTNKG ADFIATLMAV PAFSSKSSSL  300
2  ATTQKAEKEV TRMVILMVLG FMLAWTPYAV VAFWIFTNKG ADFTATLMAV PAFFSKSSSL
3  ATTQKAEKEV TRMVILMVLG FMLAWTPYAV VAFWIFTNKG ADFTATLMAV PAFFSKSSSL
4  ATTQKAEKEV TRMVILMVLG FMLAWTPYAV VAFWIFTNKG ADFTATLMSV PAFFSKSSSL

1  YNPVIYIFMN KQFRHCMITT ICCGKNPFED EDISSNIAQG KTEVSSISSS QVSPA
2  YNPIIYVLMN KQFRNCMITT ICCGKNPFGD EETSSTVSQS KTEVTSVSSS QVSPI
3  YNPIIYVLMN KQFRNCMITT ICCGKNPFGD EDVSSTVSQS KTEVSSVSSS QVSP-
4  YNPIIYVLMN KQFRNCMITT ICCGKNPFGD EEVSSTVSQS KTEVSSVSSS QVSPA
```
