# Supplementary material for: Gene loss, adaptive evolution and the co-evolution of plumage coloration genes with opsins in birds
Source: BMC Genomics. 2015 Oct 6;16:751. doi: 10.1186/s12864-015-1924-3 (PMC4595237; doi:10.1186/s12864-015-1924-3)
Supplement: Additional file 8: — Species-specific branch selection tests. The one-ratio model (H0) was tested against the two-ratios model considering the alternative hypotheses of verifying differentiated ω-ratios in the terminal lineage of the barn owl (H1.1) or the emerging lineage of penguins (H1.2). Significant alternative hypothesis are marked with a red p-value. lnL is the logarithm of the model likelihood and the LRT is the likelihood ratio test. All the LRT comparisons were performed with 1 degree of freedom and assuming a significance level of 0.05. (PDF 147 kb) [file 12864_2015_1924_MOESM8_ESM.pdf]

Gene	Lnl H0	ωb	Lnl H1.1	LRT	*p*-value	ωf	Lnl H1.2	LRT	*p*-value	ωf
RH1	-7492.031	0.043	-7490.671	2.721	0.099	0.137	-7489.300	5.464	0.019	0.119
RH2	-9263.000	0.045					-9262.362	1.276	0.259	0.091
OPN4x	-11344.030	0.155	-11343.624	0.813	0.367	0.211	-11340.842	6.376	0.012	0.546
OPN4m	-7364.207	0.196	-7363.348	1.719	0.190	0.342	-7362.636	3.142	0.076	1.337
TMT2	-4796.769	0.110	-4796.765	0.009	0.924	0.118	-4796.722	0.094	0.759	0.079
OPN3	-3662.170	0.094	-3662.052	0.237	0.627	0.065	-3661.860	0.620	0.431	0.000
PIN	-12822.260	0.146	-12806.401	31.718	0.000	0.548	-12812.983	18.554	0.000	0.527
RGR	-7675.894	0.126	-7675.623	0.543	0.461	0.181	-7675.465	0.859	0.354	0.221
RRH	-6965.018	0.134	-6963.813	2.409	0.121	0.259	-6964.692	0.653	0.419	0.061
OPN5	-8430.477	0.107	-8430.477	0.000	0.998	0.107	-8427.480	5.993	0.014	0.408
VA	-8942.038	0.236					-8941.362	1.352	0.245	0.471
TMT	-2891.969	0.211					-2890.972	1.994	0.158	0.591
