# Supplementary material for: Gene loss, adaptive evolution and the co-evolution of plumage coloration genes with opsins in birds
Source: BMC Genomics. 2015 Oct 6;16:751. doi: 10.1186/s12864-015-1924-3 (PMC4595237; doi:10.1186/s12864-015-1924-3)
Supplement: Additional file 9: — Co-evolution between opsins and the melanin-based plumage coloration genes in birds. Association tests were implemented using the ω-lineage classified in three categories: accelerated, conserved and neutral. The association between two genes was measured using the proportion of lineages showing the same evolutionary behavior and considering the alternative hypothesis p > 6/16. Values in the table correspond to the Pearson's χ2 test statistic and the sample proportions (in brackets). Significant (p-value < 8.4 x 10^−4^) and strong (p-value < 0.05) associations are indicated in red-bold and bold, respectively. (PDF 92 kb) [file 12864_2015_1924_MOESM9_ESM.pdf]

	MC1R	*TYR*	*TYRP1*	*OCA2*	*ASIP*
RH1	3.503 (0.591)	0.115 (0.340)	1.307 (0.275)	0.494 (0.308)	0.000 (0.375)
	**0.031**	0.633	0.874	0.759	0.500
RH2	10.194 (0.727)	1.304 (0.283)	0.080 (0.341)	2.533 (0.237)	1.068 (0.282)
	**0.000**	0.873	0.611	0.944	0.849
OPN1sw1		1.133 (0.529)	0.000 (0.375)		1.667 (0.188)
		0.144	0.500		0.902
OPN4m	0.303 (0.455)	2.739 (0.244)	0.667 (0.300)	0.000 (0.389)	1.186 (0.474)
	0.291	0.951	0.793	0.500	0.138
OPN4x	2.048 (0.545)	0.022 (0.354)	0.470 (0.439)	1.862 (0.256)	0.366 (0.317)
	0.076	0.559	0.247	0.914	0.727
TMT2	0.012 (0.409)	0.039 (0.349)	0.007 (0.395)	0.008 (0.353)	0.016 (0.351)
	0.456	0.578	0.467	0.535	0.551
TMT		3.267 (0.125)			
		0.965			
OPN3	0.982 (0.500)	0.022 (0.354)	0.860 (0.293)	0.002 (0.359)	0.860 (0.293)
	0.161	0.559	0.823	0.516	0.823
PIN	1.267 (0.526)	0.027 (0.350)	0.008 (0.353)	0.033 (0.406)	0.384 (0.441)
	0.130	0.565	0.535	0.428	0.268
VA	0.594 (0.273)	0.037 (0.400)	1.862 (0.256)	1.314 (0.270)	5.171 (0.564)
	0.780	0.424	0.914	0.874	**0.011**
RGR	0.109 (0.318)	1.089 (0.458)	0.080 (0.341)	0.494 (0.308)	0.366 (0.317)
	0.629	0.148	0.611	0.759	0.727
RRH	2.048 (0.545)	0.200 (0.333)	2.733 (0.512)	0.138 (0.333)	1.771 (0.488)
	0.076	0.673	**0.049**	0.645	0.092
OPN5	0.000 (0.381)	0.145 (0.413)	0.138 (0.333)	0.344 (0.316)	1.068 (0.282)
	0.500	0.352	0.645	0.721	0.849
