# Supplementary material for: Gene loss, adaptive evolution and the co-evolution of plumage coloration genes with opsins in birds
Source: BMC Genomics. 2015 Oct 6;16:751. doi: 10.1186/s12864-015-1924-3 (PMC4595237; doi:10.1186/s12864-015-1924-3)
Supplement: Additional file 11: — Avian opsin and melanin-based plumage coloration sequences. Accession numbers are indicated for the complete sequences, while for partial sequences the genomic location is given. Asterisk (*) indicate that we were not able to identify the gene in the respective genome. Gene sequences are available in the Avian Phylogenomics Project database (http://avian.genomics.cn/en/, [75]). (PDF 205 kb) [file 12864_2015_1924_MOESM11_ESM.pdf]

Species	***RH1***	***RH2***	***OPN1lw***	***OPN1sw1***	***OPN1sw2***
Acanthisitta chloris	Ach_R014274	Ach_R000688	scaffold43966	scaffold30285	scaffold10992
Anas platyrhynchos	Apl_R005227	Apl_R003993	scaffold4890	scaffold3520	scaffold4820
Apaloderma vittatum	Avi_R005355	Avi_R006088	*	scaffold41128	*
Aptenodytes forsteri	Afo_R007386	Afo_R002078	*	Scaffold310	C12507740
Balearica regulorum	Bre_R001278	scaffold11087	*	scaffold8923	scaffold39215
Buceros rhinoceros	Brh_R008392	Brh_R008642	*	scaffold38089	C11394968
Calypte anna	Aan_R004439	Aan_R003786	Aan_R011178	*	C10539872
Caprimugus carolinensis	Cca_R005137	Cca_R004539	scaffold26893	scaffold13699	*
Cariama cristata	Ccr_R004716	Ccr_R005087	*	scaffold38702	*
Cathartes aura	Cau_R009896	Cau_R006157	C11745660	scaffold64787	C11974873
Chaetura pelagica	Cpe_R007780	Cpe_R013784	scaffold672	*	scaffold1770
Charadrius vociferus	Cvo_R010602	Cvo_R006669	C14507241	scaffold904	C14818156
Chlamydotis macqueenii	Cun_R013315	Cun_R007701	*	scaffold52620	C14725356
Colius striatus	scaffold42806	Cst_R013138	C16138835	scaffold37144	scaffold54278
Columba livia	Cli_R008776	Cli_R015197	Cli_R015862	Cli_R001560	Cli_R015863
Corvus brachyrhynchos	Cbr_R016243	Cbr_R014787	scaffold807	Cbr_R004875	scaffold807
Cuculus canorus	Cca_R014544	Cca_R011163	C22900967	Cca_R007387	scaffold3131
Egretta garzetta	Ega_R008103	Ega_R000386	C7947834	scaffold358	C8058550
Eurypyga helias	Ehe_R013415	Ehe_R013243	*	C15119946	scaffold35332
Falco peregrinus	Fpe_R004520	Fpe_R005139	scaffold923_1	scaffold371_1	scaffold600_1
Fulmarus glacialis	Fgl_R000766	Fgl_R006900	*	scaffold30526	scaffold32963
Gavia stellata	Gst_R009594	Gst_R007390	*	C12403080	*
Geospiza fortis	Gfo_R002165	Gfo_R002973	scaffold2279	Gfo_R008669	C12744453
Haliaeetus albicilla	Hal_R005432	Hal_R007579	*	scaffold9295	*
Haliaeetus leucocephalus	Hle_R005132	Hle_R012171	Scaffold132	Hle_R008118	Scaffold3705
Leptosomus discolor	Ldi_R004003	Ldi_R003079	C11613766	Scaffold41452	C11468432
Manacus vitellinus	Mvi_R014802	Mvi_R013642	C10202426	Mvi_R005863	C10381930
Meleagris gallopavo	Mga_R004540	Mga_R009279	*	15	*
Melopsittacus undulatus	Mun_R006269	Mun_R003263	*	*	*
Merops nubicus	Mnu_R010479	Mnu_R007380	*	*	*
Mesitornis unicolor	Mun_R010774	Mun_R000739	scaffold22783	C13007704	scaffold42191
Nestor notabilis	Nno_R003001	Nno_R004006	scaffold27814	Nno_R004976	scaffold27473
Nipponia nippon	Nni_R014514	Nni_R010909	Scaffold2747	Nni_R013924	Scaffold2747
Opisthocomus hoazin	Oho_R001071	Oho_R001168	*	scaffold1001	*
Pelecanus crispus	Pcr_R007634	Pcr_R007325	*	C6960666	*
Phaethon lepturus	Ple_R011293	Ple_R005725	*	scaffold27297	*
Phalacrocorax carbo	Pca_R000096	Pca_R001029	*	*	*
Phoenicopterus ruber	Pru_R011144	Pru_R006483	*	C16030346	*
Picoides pubescens	Ppu_R002287	Ppu_R009745	C40097764	Ppu_R009312	Ppu_R003169
Podiceps cristatus	Pcr_R005418	Pcr_R008848	*	scaffold36774	*
Pterocles gutturalis	Pgu_R008658	Pgu_R012270	scaffold18039	scaffold34007	scaffold14537
Pygoscelis adeliae	Pad_R007548	Pad_R004695	C9772274	C9752044	*
Struthio camelus	Sca_R016073	Sca_R011489	*	*	scaffold1886
Tauraco erythrolophus	Ter_R010357	Ter_R006249	*	scaffold30365	*
Tinamus guttatus	Tma_R011592	Tma_R007266	C15395113	Tma_R005000	scaffold13658
Tyto alba	Tal_R010820	Tal_R007273	*	*	*

Species	***OPN4m***	***OPN4x***	***TMT2***	***TMT***	***OPN3***
Acanthisitta chloris	Ach_R001224	Ach_R001141	Ach_R012812	*	Ach_R005051
Anas platyrhynchos	Apl_R003228	Apl_R001728	Apl_R006312	*	Apl_R000897
Apaloderma vittatum	Avi_R003920	Avi_R001566	Avi_R010131	*	Avi_R006711
Aptenodytes forsteri	Afo_R000425	Afo_R015633	Afo_R015920	Afo_R009918	Afo_R000248
Balearica regulorum	Bre_R011216	Bre_R009736	Bre_R000730	scaffold519	Bre_R000424
Buceros rhinoceros	Brh_R003376	Brh_R009958	Brh_R004335	*	Brh_R008074
Calypte anna	Aan_R009582	Aan_R005208	Aan_R003171	*	Aan_R007013
Caprimugus carolinensis	Cca_R013356	Cca_R013105	Cca_R012283	Cca_R004789	Cca_R008857
Cariama cristata	Ccr_R002694	Ccr_R011020	Ccr_R012244	*	Ccr_R012148
Cathartes aura	Cau_R007941	Cau_R013353	Cau_R002259	scaffold43101	Cau_R012651
Chaetura pelagica	Cpe_R008180	Cpe_R005108	Cpe_R001898	*	Cpe_R006702
Charadrius vociferus	Cvo_R010420	Cvo_R010129	Cvo_R016531	Cvo_R013965	Cvo_R007685
Chlamydotis macqueenii	Cun_R000826	Cun_R012921	Cun_R012345	*	Cun_R003358
Colius striatus	Cst_R006583	Cst_R007760	Cst_R012706	*	Cst_R001751
Columba livia	Cli_R009647	Cli_R007017	Cli_R005022	*	Cli_R005630
Corvus brachyrhynchos	Cbr_R007418	Cbr_R006170	Cbr_R015319	Cbr_R006030	Cbr_R013263
Cuculus canorus	Cca_R011740	Cca_R006810	Cca_R007498	*	Cca_R007991
Egretta garzetta	scaffold280	Ega_R014927	Ega_R011783	Ega_R002951	scaffold248
Eurypyga helias	Ehe_R001763	Ehe_R002676	Ehe_R001690	Ehe_R003531	Ehe_R012682
Falco peregrinus	Fpe_R008091	Fpe_R012248	Fpe_R016144	*	Fpe_R005610
Fulmarus glacialis	Fgl_R011238	Fgl_R008649	Fgl_R012037	Fgl_R011481	Fgl_R006359
Gavia stellata	Gst_R002007	Gst_R004211	Gst_R009280	Gst_R002112	Gst_R011107
Geospiza fortis	Gfo_R002543	Gfo_R000912	Gfo_R005581	Gfo_R000463	Gfo_R003748
Haliaeetus albicilla	Hal_R002915	Hal_R004749	Hal_R000785	*	Hal_R001996
Haliaeetus leucocephalus	Hle_R000301	Hle_R003722	Hle_R003707	*	Hle_R010883
Leptosomus discolor	Ldi_R003466	Ldi_R007922	Ldi_R012455	Ldi_R011148	scaffold28918
Manacus vitellinus	Mvi_R004971	Mvi_R008071	Mvi_R004631	*	Mvi_R000726
Meleagris gallopavo	Mga_R013741	Mga_R010815	Mga_R001808	*	Mga_R006440
Melopsittacus undulatus	Mun_R013934	Mun_R009058	Mun_R011665	*	Mun_R006485
Merops nubicus	Mnu_R005078	Mnu_R013336	Mnu_R002741	*	Mnu_R000042
Mesitornis unicolor	Mun_R010255	Mun_R003555	Mun_R004968	Mun_R001569	Mun_R006478
Nestor notabilis	Nno_R013303	Nno_R005879	Nno_R008442	*	Nno_R013820
Nipponia nippon	Nni_R012773	Nni_R001008	Nni_R002849	Nni_R001657	Nni_R000220
Opisthocomus hoazin	Oho_R002541	Oho_R002893	Oho_R014763	*	Oho_R002783
Pelecanus crispus	Pcr_R007180	Pcr_R008160	Pcr_R004060	Pcr_R014396	Pcr_R001432
Phaethon lepturus	Ple_R009482	Ple_R011463	Ple_R001502	scaffold43492	Ple_R000756
Phalacrocorax carbo	Pca_R002233	Pca_R009405	Pca_R002610	scaffold4977	Pca_R012581
Phoenicopterus ruber	Pru_R013367	Pru_R004016	Pru_R011480	scaffold29446	Pru_R005042
Picoides pubescens	Ppu_R009502	Ppu_R007138	Ppu_R009199	Ppu_R006252	Ppu_R003550
Podiceps cristatus	Pcr_R011580	Pcr_R010356	Pcr_R012678	Pcr_R004160	Pcr_R007428
Pterocles gutturalis	Pgu_R008215	Pgu_R004019	Pgu_R008581	*	scaffold52099
Pygoscelis adeliae	Pad_R008885	Pad_R013289	Pad_R003148	Pad_R014502	Pad_R014030
Struthio camelus	scaffold310	Sca_R006216	Sca_R014327	Sca_R010905	scaffold367
Tauraco erythrolophus	Ter_R010818	Ter_R002755	Ter_R015046	*	Ter_R014568
Tinamus guttatus	Tma_R001937	Tma_R008516	Tma_R002341	*	Tma_R010398
Tyto alba	Tal_R011509	Tal_R001976	Tal_R013018	*	Tal_R007498

Species	***PIN***	***VA***	***RGR***	***RRH***	***OPN5***
Acanthisitta chloris	C16157070	Ach_R011655	Ach_R013917	Ach_R003910	XM_009081264.1
Anas platyrhynchos	Apl_R005168	Apl_R014177	Apl_R003214	Apl_R014026	XM_005012930.1
Apaloderma vittatum	Avi_R013589	Avi_R005681	Avi_R001147	Avi_R002418	XM_009873315.1
Aptenodytes forsteri	Afo_R013563	Afo_R002916	Afo_R001551	Afo_R015696	XM_009273858.1
Balearica regulorum	Bre_R000859	Bre_R000642	Bre_R001897	Bre_R005316	XM_010302599.1
Buceros rhinoceros	Brh_R013509	Brh_R011866	Brh_R011273	Brh_R002677	XM_010142294.1
Calypte anna	Aan_R011213	Aan_R010452	Aan_R012485	Aan_R005179	XM_008490719.1
Caprimugus carolinensis	Cca_R006665	Cca_R011029	Cca_R001020	Cca_R013387	XM_010174807.1
Cariama cristata	Ccr_R004711	Ccr_R007095	Ccr_R006703	Ccr_R004757	XM_009701094.1
Cathartes aura	*	Cau_R007663	Cau_R006452	Cau_R011607	scaffold40501
Chaetura pelagica	Cpe_R013541	Cpe_R004165	Cpe_R008196	Cpe_R014667	XM_010002392.1
Charadrius vociferus	Cvo_R015435	Cvo_R009442	Cvo_R010435	Cvo_R012588	XM_009885296.1
Chlamydotis macqueenii	Cun_R011675	Cun_R009552	Cun_R001574	Cun_R006475	XM_010120848.1
Colius striatus	Cst_R006686	Cst_R010103	Cst_R011582	Cst_R009961	XM_010199969.1
Columba livia	Cli_R012115	Cli_R001640	Cli_R012948	Cli_R016297	XM_005510202.1
Corvus brachyrhynchos	Cbr_R007748	Cbr_R012082	Cbr_R007434	Cbr_R004289	XM_008641820.1
Cuculus canorus	Cca_R012507	Cca_R010500	Cca_R011757	Cca_R001866	XM_009558441.1
Egretta garzetta	Ega_R000861	Ega_R015166	Ega_R006443	Ega_R010020	XM_009639608.1
Eurypyga helias	Ehe_R012817	Ehe_R008692	Ehe_R003031	Ehe_R012194	XM_010149210.1
Falco peregrinus	*	Fpe_R008757	Fpe_R008106	Fpe_R006767	XM_005239189.1
Fulmarus glacialis	*	Fgl_R013036	Fgl_R011993	Fgl_R001878	XM_009587844.1
Gavia stellata	*	Gst_R002069	Gst_R009355	Gst_R001159	XM_009817945.1
Geospiza fortis	Gfo_R012798	Gfo_R008627	Gfo_R002528	Gfo_R000958	XM_005418407.1
Haliaeetus albicilla	Hal_R001793	Hal_R013789	Hal_R003830	Hal_R012291	XM_009915908.1
Haliaeetus leucocephalus	Hle_R010722	Hle_R008545	Hle_R010002	Hle_R007366	XM_010584279.1
Leptosomus discolor	Ldi_R005008	Ldi_R002540	Ldi_R005745	Ldi_R001421	XM_009948078.1
Manacus vitellinus	Mvi_R015224	Mvi_R001566	Mvi_R006107	Mvi_R000585	XM_008927044.1
Meleagris gallopavo	Mga_R007839	Mga_R014261	Mga_R013723	Mga_R011100	XM_010708184.1
Melopsittacus undulatus	Mun_R003348	Mun_R004570	Mun_R013919	Mun_R009000	XM_005146432.1
Merops nubicus	Mnu_R002284	Mnu_R006973	Mnu_R000635	Mnu_R005172	XM_008940418.1
Mesitornis unicolor	Mun_R001769	Mun_R011983	Mun_R014433	Mun_R005332	XM_010178658.1
Nestor notabilis	Nno_R008673	Nno_R002048	Nno_R009491	Nno_R001823	Nno_R013070
Nipponia nippon	Nni_R006596	Nni_R013156	Nni_R012758	Nni_R000942	XM_009467326.1
Opisthocomus hoazin	Oho_R012156	Oho_R004817	Oho_R001256	Oho_R000992	XM_009937828.1
Pelecanus crispus	Pcr_R006937	Pcr_R012292	Pcr_R012529	Pcr_R013678	XM_009492185.1
Phaethon lepturus	Ple_R010350	Ple_R012232	Ple_R002415	Ple_R006613	XM_010292092.1
Phalacrocorax carbo	Pca_R004459	Pca_R009809	Pca_R008814	Pca_R010769	XM_009500831.1
Phoenicopterus ruber	Pru_R000649	Pru_R006632	Pru_R012832	Pru_R009308	scaffold19272
Picoides pubescens	Ppu_R007334	Ppu_R001292	Ppu_R009519	Ppu_R012373	XM_009905310.1
Podiceps cristatus	Pcr_R006037	scaffold55602	Pcr_R002939	Pcr_R004406	Pcr_R011050
Pterocles gutturalis	Pgu_R009989	Pgu_R004550	Pgu_R007305	Pgu_R011442	XM_010086910.1
Pygoscelis adeliae	Pad_R007184	Pad_R009724	Pad_R008871	Pad_R008435	XM_009319665.1
Struthio camelus	*	Sca_R002519	Sca_R007528	Sca_R012133	XM_009667941.1
Tauraco erythrolophus	Ter_R015417	Ter_R001995	Ter_R014106	Ter_R013837	XM_009982410.1
Tinamus guttatus	*	scaffold2217	Tma_R004075	Tma_R010431	XM_010220590.1
Tyto alba	Tal_R006759	scaffold7106	Tal_R004924	Tal_R013125	XM_009971991.1

Species	***OCA2***	***ASIP***	***TYR***	***TYRP1***	***MC1R***
Acanthisitta chloris	Ach_R001753	scaffold19384	Ach_R008651	Ach_R010336	Ach_R010237
Anas platyrhynchos	Apl_R001901	Apl_R010739	Apl_R007032	scaffold363	Apl_R012273
Apaloderma vittatum	scaffold6063	Avi_R002363	Avi_R003613	Avi_R007091	C11067011
Aptenodytes forsteri	Afo_R005363	Afo_R008324	Afo_R009840	Afo_R010783	Afo_R007528
Balearica regulorum	Bre_R013695	Bre_R004216	Bre_R006844	scaffold3007	scaffold34776
Buceros rhinoceros	Brh_R013403	scaffold37575	Brh_R000792	Brh_R003616	scaffold36017
Calypte anna	Aan_R003232	Aan_R010843	Aan_R001745	Aan_R015578	scaffold330
Caprimugus carolinensis	Cca_R003164	Cca_R012605	Cca_R006373	Cca_R012949	scaffold32048
Cariama cristata	Ccr_R011914	Ccr_R011875	Ccr_R001243	Ccr_R012153	C10354424
Cathartes aura	Cau_R013460	Cau_R010314	Cau_R007049	Cau_R011421	Cau_R006551
Chaetura pelagica	Cpe_R006175	scaffold1032	Cpe_R007882	Cpe_R008143	Cpe_R005496
Charadrius vociferus	Cvo_R006810	Cvo_R010076	Cvo_R000148	Cvo_R004648	Cvo_R008983
Chlamydotis macqueenii	Cun_R001729	Cun_R004370	Cun_R005445	Cun_R013118	scaffold43988
Colius striatus	scaffold12027	Cst_R006539	Cst_R002549	Cst_R003325	scaffold51532
Columba livia	Cli_R007350	Cli_R016011	Cli_R012403	Cli_R012418	Cli_R001740
Corvus brachyrhynchos	Cbr_R015986	Cbr_R010651	Cbr_R010862	Cbr_R004835	Cbr_R009703
Cuculus canorus	Cca_R007812	Cca_R001462	Cca_R002034	Cca_R009722	Cca_R010640
Egretta garzetta	Ega_R015319	Ega_R005289	Ega_R001847	scaffold175	scaffold428
Eurypyga helias	Ehe_R013742	Ehe_R000869	Ehe_R011924	Ehe_R001948	C14857594
Falco peregrinus	Fpe_R016211	Fpe_R015508	Fpe_R012499	Fpe_R003044	Fpe_R009326
Fulmarus glacialis	scaffold14041	Fgl_R002741	Fgl_R011593	Fgl_R000634	scaffold31831
Gavia stellata	Gst_R001151	Gst_R012923	Gst_R002927	Gst_R001758	C12114711
Geospiza fortis	Gfo_R009685	Gfo_R011672	Gfo_R003679	Gfo_R001937	Gfo_R000540
Haliaeetus albicilla	scaffold19972	Hal_R000525	Hal_R006345	Hal_R008176	*
Haliaeetus leucocephalus	Hle_R015907	Hle_R011017	Hle_R016207	Hle_R016466	Hle_R009468
Leptosomus discolor	Ldi_R002849	scaffold8251	Ldi_R008956	Ldi_R011186	C11262141
Manacus vitellinus	Mvi_R008334	Mvi_R005301	Mvi_R012210	Mvi_R002298	Mvi_R004812
Meleagris gallopavo	Mga_R001749	Mga_R007970	Mga_R002335	Z	Mga_R004146
Melopsittacus undulatus	Mun_R011728	Mun_R007921	Mun_R010012	Mun_R015408	Mun_R000562
Merops nubicus	Mnu_R005282	scaffold19071	Mnu_R002069	Mnu_R001084	Mnu_R000150
Mesitornis unicolor	Mun_R010375	Mun_R005658	Mun_R015332	Mun_R006435	Mun_R008222
Nestor notabilis	scaffold8246	Nno_R002042	Nno_R000837	Nno_R003435	Nno_R002444
Nipponia nippon	Nni_R002782	*	Nni_R013463	Nni_R004747	Nni_R011019
Opisthocomus hoazin	Oho_R014279	Oho_R012825	Oho_R000102	Oho_R009777	scaffold2471
Pelecanus crispus	scaffold15426	Pcr_R013745	Pcr_R001051	scaffold3980	scaffold25530
Phaethon lepturus	Ple_R001146	Ple_R002840	Ple_R000620	Ple_R012694	C12929869
Phalacrocorax carbo	scaffold8622	Pca_R003876	Pca_R006344	Pca_R012291	scaffold44024
Phoenicopterus ruber	scaffold5937	Pru_R010194	Pru_R013834	Pru_R000806	scaffold20554
Picoides pubescens	Ppu_R015571	Ppu_R002601	Ppu_R009434	Ppu_R002577	Ppu_R003649
Podiceps cristatus	Pcr_R007255	scaffold676	Pcr_R009349	Pcr_R005586	C16189223
Pterocles gutturalis	Pgu_R012705	Pgu_R008305	Pgu_R004255	Pgu_R012109	scaffold5252
Pygoscelis adeliae	Pad_R002840	Pad_R013742	Pad_R002612	Pad_R010951	C9919992
Struthio camelus	Sca_R007482	Sca_R012191	Sca_R004770	scaffold13	scaffold1461
Tauraco erythrolophus	Ter_R000600	Ter_R012741	Ter_R005801	Ter_R004022	Ter_R008331
Tinamus guttatus	Tma_R002862	Tma_R003505	Tma_R010061	Tma_R003004	scaffold1475
Tyto alba	scaffold16050	Tal_R010508	Tal_R012303	scaffold13067	*
