# Supplementary material for: Gene loss, adaptive evolution and the co-evolution of plumage coloration genes with opsins in birds
Source: BMC Genomics. 2015 Oct 6;16:751. doi: 10.1186/s12864-015-1924-3 (PMC4595237; doi:10.1186/s12864-015-1924-3)
Supplement: Additional file 12: — Avian sw2 and lw sequences. Accession number of the sw2 and lw sequences used for site selection analyses. (PDF 12 kb) [file 12864_2015_1924_MOESM12_ESM.pdf]

Gene	Species	Acession number
OPN1lw	*Gallus gallus*	NM_205440.1
OPN1lw	*Platycercus elegans*	KF134493.1
OPN1lw	*Taeniopygia guttata*	NM_001076702.1
OPN1lw	*Ptilonorhynchus violaceus*	JQ034370.1
OPN1lw	*Ailuroedus crassirostris*	JQ034368.1
OPN1lw	*Scenopoeetes dentirostris*	JQ034369.1
OPN1lw	*Chlamydera maculata*	JQ034372.1
OPN1lw	*Serinus canaria*	AJ277925.1
OPN1lw	*Columba livia*	XM_005514006.1
OPN1lw	*Pseudopodoces humilis*	XM_005534472.1
OPN1lw	*Prionodura newtoniana*	JQ218138.1
OPN1lw	*Scenopoeetes dentirostris*	JQ034369.1
OPN1lw	*Falco cherrug*	XM_005436734.1
OPN1lw	*Calypte anna*	XM_008500645.1
OPN1lw	*Chlamydera nuchalis*	JQ034373.1
OPN1lw	*Chlamydera cerviniventris*	JQ218135.1
OPN1lw	*Sericulus chrysocephalus*	JQ034371.1
OPN1sw2	*Columba livia*	NM_001310048.1
OPN1sw2	*Gallus gallus*	NM_205517.1
OPN1sw2	*Pseudopodoces humilis*	XM_005534405.1
OPN1sw2	*Taeniopygia guttata*	NM_001076697.1
OPN1sw2	*Ptilonorhynchus violaceus*	JQ034388.1
OPN1sw2	*Chlamydera maculata*	JQ034390.1
OPN1sw2	*Ailuroedus crassirostris*	JQ034386.1
OPN1sw2	*Serinus canaria*	AJ277923.1
OPN1sw2	*Platycercus elegans*	KF134491.1
OPN1sw2	*Sericulus chrysocephalus*	JQ034389.1
OPN1sw2	*Picoides pubescens*	XM_009898807.1
OPN1sw2	*Chlamydera nuchalis*	JQ034391.1
OPN1sw2	*Scenopoeetes dentirostris*	JQ034387.1
