# Supplementary material for: Gene loss, adaptive evolution and the co-evolution of plumage coloration genes with opsins in birds
Source: BMC Genomics. 2015 Oct 6;16:751. doi: 10.1186/s12864-015-1924-3 (PMC4595237; doi:10.1186/s12864-015-1924-3)
Supplement: Additional file 13: — Mammalian opsin sequences. Accession number of the mammalian opsins sequences used for site selection analyses. (PDF 88 kb) [file 12864_2015_1924_MOESM13_ESM.pdf]

Species	*RH1*	*OPN1sw1*	*OPN1lw*	*OPN3*	*OPN5*	*RGR*	*RRH*	*OPN4m*
Ornithorhynchus_anatinus	NM_001127627.1		EF050078.1		XM_007667004.1	XM_007657671.1	XM_001506366.2	XM_007660572.1
Tachyglossus_aculeatus	JX103830.1		EU636011.1					
Zaglossus_bruijni			DQ111869.1					
Myrmecobius_fasciatus		AY726546.2	DQ111870.1					
Sarcophilus_harrisii	XM_003762449.1	XM_003771592.2	XM_003774721.2	XM_003767787.1	XM_003769136.1		XM_003774474.2	XM_003754913.1
Sminthopsis_crassicaudata	AY159786.2	AY442173.1	AY430816.1					DQ383281.1
Monodelphis_domestica	XM_001366188.2	XM_007504107.1	NM_001145081.1	XM_001377887.2	XM_007484056.1		XM_007495995.1	XM_007478037.1
Caluromys_philander	AY313946.1							
Didelphis_aurita		DQ352182.1	DQ352180.1					
Thylamys_elegans		DQ356245.1	DQ356244.1					
Cercartetus_concinnus		AY772473.1	AY772471.1					
Macropus_eugenii		AY286017.1	AY286018.1					
Setonix_brachyurus		AY726545.1	AY745192.1					
Tarsipes_rostratus		AY772472.1	AY772470.1					
Isoodon_obesulus		AY726544.2	AY745193.1					
Dasypus_novemcinctus	XM_004477246.1			XM_012522085.1	XM_004452792.1	XM_004484497.2	XM_012518661.1	XM_004483219.1
Choloepus_didactylus	AB455169.1							
Myrmecophaga_tridactyla	AB455189.1							
Tamandua_tetradactyla	AB455203.1							
Amblysomus_hottentotus	GQ290298.1							
Chrysochloris_asiatica	XM_006868670.1	XM_006861111.1			XM_006860534.1		XM_006869316.1	XM_006875926.1
Echinops_telfairi	XM_004702378.1	XM_004707803.1	XM_004717562.1		XM_004696371.1	XM_004713040.1	XM_004703460.1	XM_013006313.1
Procavia_capensis	AB455196.1							
Elephantulus_edwardii	XM_006901040.1	XM_006896064.1	XM_006899926.1		XM_006881924.1	XM_006901024.1	XM_006881091.1	XM_006894487.1
Macroscelides_proboscideus	GQ290310.1							
Elephas_maximus	AB455174.1							
Loxodonta_africana	NM_001280858.1	NM_001280859.1	NM_001280862.1	XM_003410915.2	XM_003404446.2	XM_003418559.1	XM_003410397.1	XM_003418564.1
Dugong_dugon	AB455173.1							
Trichechus_manatus	AF055319.1		XM_004390580.1	XM_004375853.1	XM_004379536.1	XM_004389893.1	XM_004380256.2	XM_004386107.2
Orycteropus_afer_afer	XM_007956743.1	XM_007955413.1	XM_007957152.1	XM_007942718.1	XM_007936162.1	XM_007954235.1	XM_007940751.1	XM_007955933.1
Ailuropoda_melanoleuca	XM_002921249.2	XM_002913423.2	XM_002928811.2	XM_011223981.1	XM_002919006.1	XM_002915389.2	XM_002929340.1	XM_011219945.1
Artibeus_jamaicensis	GQ290299.1							
Balaenoptera_acutorostrata	XM_007192608.1	XM_007177192.1	XM_007185533.1	XM_007182615.1	XM_007193850.1	XM_007174576.1	XM_007191029.1	XM_007174566.1
Bison_bison	XM_010862448.1	XM_010831706.1	XM_010840278.1		XM_010863044.1	XM_010854377.1	XM_010858539.1	XM_010854385.1
Bos_mutus	XM_005902834.1	XM_005905247.1	XM_005909080.1		XM_005904414.1	XM_005902591.1	XM_005896227.1	XM_005909983.1
Bos_taurus	NM_001014890.1	NM_174567.1	NM_174566.1		XM_010818222.1	NM_175775.2	NM_001192224.1	NM_001192399.1
Bubalus_bubalis	XM_006078900.1	XM_006074392.1	XM_006043645.1		XM_006076114.1	XM_006072363.1	XM_006052204.1	XM_006066606.1
Camelus_bactrianus	XM_010953086.1	XM_010947561.1	XM_010964076.1	XM_010974055.1		XM_010963086.1	XM_010953677.1	XM_010953736.1
Camelus_dromedarius	XM_010984900.1	XM_010975637.1	XM_010991460.1	XM_010992162.1	XM_010995432.1	XM_010984078.1		XM_010997691.1
Camelus_ferus	XM_006180073.1	XM_006184725.1	XM_006188804.1	XM_006195663.1	XM_006190729.1	XM_006173999.1	XM_006188932.1	XM_006194367.1
Canis_lupus	XM_005632035.1	XM_539386.3	NM_001197072.1	XM_005622576.1	XM_003639428.2	XM_546190.4	XM_005639270.1	XM_005619440.1
Capra_hircus		XM_005679445.1	XM_005701738.1		XM_005696391.1	XM_005699305.1	XM_005681311.1	XM_005699313.1
Ceratotherium_simum	XM_004442424.1	XM_004418710.1	XM_004443325.1	XM_004439605.1	XM_004424042.1	XM_004432483.1	XM_004426582.1	XM_004432477.1
Chaerephon_plicatus	XM_004692360.2	XM_004677016.2	XM_012734472.1				XM_004686475.1	
Cynopterus_sphinx	GQ863420.1	GQ863415.1	GQ863439.1					
Delphinus_delphis	AF055314.1		AY228451.1					
Enhydra_lutris	AY883931.1		AY884350.1					
Eptesicus_fuscus	XM_008152292.1	XM_008154038.1	XM_008159259.1		XM_008153205.1	XM_008149130.1	XM_008150280.1	XM_008149131.1
Equus_caballus	XM_001490301.3	XM_001502735.4	AF031531.1	XM_005607945.1	XM_001502775.2	XM_001495788.2	XM_001503520.3	XM_005602742.1
Erignathus_barbatus	AY883932.1		AY884351.1					
Erinaceus_europaeus	XM_007517079.1	XM_007526304.1	XM_007534857.1		XM_007517838.1	XM_007533920.1	XM_007534492.1	XM_007530217.1
Eubalaena_glacialis	JQ730751.1							
Felis_catus	NM_001009242.1	XM_003983019.3	AF031532.1	XM_003999138.3	XM_003986222.2	XM_003994126.2	XM_003985084.1	XM_006937944.1
Globicephala_melas	AF055315.1		AY228446.1					
Haplonycteris_fischeri		AM263196.1	AM263193.1					
Hipposideros_armiger	GQ863417.1		GQ863448.1					
Hipposideros_larvatus	GQ290308.1		GQ863445.1					
Hipposideros_pomona	GQ863426.1		GQ863454.1					
Hipposideros_pratti	GQ290309.1							
Hydrurga_leptonyx	AY883930.1							
Leptonychotes_weddellii	XM_006740235.1	XM_006745503.1	XM_006739353.1	XM_006746683.1	XM_006727572.1	XM_006742966.1	XM_006727200.1	XM_006740386.1
Megaderma_spasma	GQ290316.1							
Mesoplodon_bidens	AF055316.1							
Miniopterus_fuliginosus	GQ863431.1	GQ863406.1	GQ863447.1					
Mirounga_angustirostris	AY228452.1		AY884347.1					
Mustela_putorius	XM_004738577.1	XM_004789507.1	XM_004780711.1	XM_004756757.1	XM_004739727.1	XM_004765768.1	XM_004748079.1	XM_004765783.1
Myotis_brandtii	XM_005870029.1	XM_005868920.1	XM_005886120.1		XM_005862327.1	XM_005871152.1	XM_005872403.1	XM_005871127.1
Myotis_davidii	XM_006758300.1	XM_006771393.1	GQ863453.1		XM_006765717.1	XM_006755598.1	XM_006755231.1	XM_006767920.1
Myotis_laniger	GQ863435.1	GQ863407.1	GQ863442.1					
Myotis_lucifugus	XM_006083811.1	GQ225742.1	GQ225743.1		XM_006093215.1	XM_006100132.1	XM_006101369.1	XM_006100103.1
Myotis_ricketti	GQ290312.1	EU662250.1	GQ863444.1					
Myotis_velifer		AM263197.1	AM263194.1					

Species	***RH1***	***OPN1sw1***	***OPN1lw***	***OPN3***	***OPN5***	***RGR***	***RRH***	***OPN4m***
Nyctalus_plancyi	GQ863422.1	GQ863408.1	GQ863459.1					
Odobenus_rosmarus	XM_004395657.1	XM_004398023.2	AY884344.1	XM_004414269.1	XM_004408452.1	XM_004416238.1	XM_004411253.2	XM_004410203.1
Odocoileus_virginianus			AF132041.1					
Orcinus_orca	XM_004284305.2	XM_004272260.1	XM_004286442.2	XM_004283495.1	XM_004267503.1	XM_004273014.1	XM_004269577.1	XM_004273009.1
Ovis_aries	XM_004018534.2	XM_012131493.1	XM_004022260.2		XM_012114096.1	XM_004021531.2	XM_004009620.2	XM_012152559.1
Panthera_tigris	XM_007075434.1	XM_007078061.1		XM_007077038.1	XM_007085094.1	XM_007093130.1	XM_007098257.1	XM_007093138.1
Pantholops_hodgsonii	XM_005955745.1	XM_005981411.1	XM_005957735.1		XM_005960627.1	XM_005972724.1	XM_005970518.1	XM_005964475.1
Phoca_groenlandica	AF055318.1		AY228449.1					
Phoca_hispida	AY883927.1		AY884346.1					
Phoca_vitulina	AF055317.1		AF110495.1					
Phocoena_phocoena			AY228450.1					
Physeter_catodon	XM_007126220.1	XM_007119765.1	XM_007113595.1	XM_007130878.1	XM_007127388.1	XM_007104038.1	XM_007105856.1	XM_007120485.1
Pteropus_alecto	XM_006917646.1	XM_006910537.1	XM_006915297.1	XM_006924119.1	XM_006926469.1	XM_006915458.1	XM_006919063.1	XM_006915451.1
Pteropus_dasymallus		AM263192.1	AM263195.1					
Pteropus_vampyrus	XM_011370659.1	XM_011357263.1	XM_011375552.1	XM_011380004.1	XM_011354795.1	XM_011355342.1	XM_011361528.1	XM_011355352.1
Rangifer_tarandus		FN808318.1						
Rhinolophus_ferrumequinum	GQ290314.1							
Rhinolophus_pearsonii	GQ863432.1		GQ863457.1					
Rhinolophus_pusillus	GQ290315.1		GQ863452.1					
Rhinolophus_sinicus	GQ863434.1	GQ863413.1	GQ863446.1					
Rousettus_leschenaultii	GQ290317.1		GQ863440.1					
Sorex_araneus	XM_004613232.1	XM_004608279.2	XM_004606597.1		XM_012932158.1	XM_004607557.1	XM_004612758.1	XM_012932474.1
Sus_scrofa	NM_214221.1	NM_214090.1	NM_001011506.1		XM_005674065.1	XM_005671156.1	XM_003129266.1	XM_001925749.2
Taphozous_melanopogon	GQ290318.1	GQ863411.1	GQ863443.1					
Tursiops_truncatus	NM_001280659.1		AF055457.1		XM_004314422.1	XM_004320073.1	XM_004330526.1	XM_004324747.1
Ursus_maritimus	XM_008697847.1	XM_008705848.1	AY884345.1	XM_008697245.1	XM_008691820.1	XM_008696318.1	XM_008689511.1	XM_008696127.1
Vicugna_pacos	XM_006206787.1	XM_006202271.1	XM_006218499.1	XM_006220133.1	XM_006201926.1	XM_006213095.1	XM_006212047.1	XM_006217382.1
Zalophus_californianus	AY883924.1		AY884343.1					
Alouatta_palliata			AB809459.1					
Alouatta_pigra			AB809461.1					
Alouatta_seniculus			AH007056.2					
Arvicanthis_ansorgei	EU862075.1							KC150901.1
Ateles_geoffroyi			AB193790.1					
Callithrix_jacchus	XM_008982215.1	XM_002752031.2	AB046546.1	XM_003735287.2	XM_002746623.2	XM_009009624.1	XM_002745490.3	XM_009009611.1
Cavia_porcellus	NM_001173085.1	NM_001172758.1	AF031529.1	XM_003474311.2	XM_003473618.1	XM_003466148.1	XM_003467998.1	XM_003466144.1
Cebus_capucinus			AB193772.1					
Cebus_olivaceus			AH006290.2					
Cercocebus_atys	XM_012060295.1	XM_012087993.1	XM_012088314.1	XM_012036274.1	XM_012070473.1	XM_012077132.1	XM_012029027.1	XM_012077116.1
Chinchilla_lanigera	XM_005387085.1	XM_005402383.1	XM_005414184.1	XM_005374819.1	XM_005389879.1	XM_005407883.1	XM_005406281.1	
Chlorocebus_sabaeus	XM_007985378.1	XM_007982851.1	XM_007993132.1	XM_007989937.1	XM_007972311.1		XM_007999533.1	XM_007962704.1
Colobus_angolensis	XM_011950692.1	XM_011939561.1		XM_011955692.1	XM_011960271.1	XM_011950422.1	XM_011945758.1	XM_011950413.1
Cricetulus_griseus	NM_001244407.1	XM_003497518.2	XM_003506533.2	XM_007639594.1	XM_007626739.1	XM_003504562.1	XM_003515437.2	XM_003495335.2
Fukomys_damarensis	XM_010637946.1	XM_010633453.1	XM_010628046.1	XM_010642658.1	XM_010617783.1	XM_010622597.1	XM_010641519.1	XM_010622583.1
Galeopterus_variegatus	XM_008574046.1	XM_008585390.1	XM_008578409.1	XM_008575257.1	XM_008586585.1	XM_008586092.1	XM_008570183.1	XM_008570316.1
Gorilla_gorilla	XM_004036292.1	AH005811.2	XM_004065128.1	XM_004028670.1	XM_004044155.1	XM_004049701.1	XM_004040277.1	XM_004049711.1
Heterocephalus_glaber	XM_004902702.1	XM_004856389.1		XM_004921366.1	XM_004846396.1	XM_004891582.1	XM_004878833.1	XM_004878214.1
Homo_sapiens	KJ849294.1	NM_001708.2	CR749814.1	AF482426.1	XM_011514347.1		BC128401.1	AB065730.1
Jaculus_jaculus	XM_004651581.1	XM_004661221.2	XM_004672101.2	XM_004653438.1	XM_004649433.2	XM_004658006.1	XM_004658625.1	XM_004657836.1
Lagothrix_lagotricha			AB467314.1					
Macaca_fascicularis	NM_001283360.1	NM_001283204.1	AF158968.1	XM_005539667.1	XM_005552779.1	XM_005565268.1	XM_005555667.1	XM_005565254.1
Macaca_mulatta	XM_001094250.1	XM_001091869.2		XM_001094239.2	XM_001104044.2	XM_001086190.2	XM_002808418.1	XM_001088248.2
Macaca_nemestrina	XM_011734526.1	XM_011729315.1	XM_011716774.1	XM_011729473.1	XM_011756883.1	XM_011766017.1	XM_011749030.1	XM_011765995.1
Mandrillus_leucophaeus	XM_011974200.1	XM_011995905.1	XM_012002287.1	XM_011990032.1	XM_011986880.1	XM_011985817.1	XM_011970568.1	XM_011995396.1
Mesocricetus_auratus	XM_005066100.1		XM_005086920.1	XM_005078169.1	XM_005072296.1	XM_005077615.1	XM_005082830.1	XM_005077694.1
Microcebus_murinus						XM_012764714.1		
Microtus_ochrogaster	XM_005365058.1	XM_005365745.1	XM_005370706.1	XM_005368525.1	XM_005359789.1	XM_005348668.1	XM_005357272.1	XM_005348669.1
Miopithecus_talapoin								
Mus_musculus	AK044412.1		NM_008106.2	AF482427.1	XM_006524455.2		AK021379.1	BC139827.1
Nannospalax_ehrenbergi	AF309568.1		AF139726.1					AM748539.1
Nannospalax_galili	XM_008854920.1	XM_008846382.1	XM_008829579.1	XM_008849180.1	XM_008846735.1	XM_008835592.1	XM_008843722.1	XM_008824437.1
Nomascus_leucogenys	XM_003265030.3	XM_003261297.2	XM_003279299.1	XM_012506427.1	XM_003254193.1	XM_012502904.1	XM_003269355.3	XM_003278589.1
Ochotona_princeps	XM_004581320.1	XM_004592516.1	XM_004598859.1	XM_004578507.2	XM_004590534.1	XM_004583348.1	XM_004594526.1	XM_012926407.1
Octodon_degus	XM_004645617.2	XM_004642726.1	XM_004645096.2	XM_004626818.1	XM_004624089.2	XM_004641249.1	XM_004626684.1	XM_012516915.1
Oryctolagus_cuniculus	XM_008260202.1	XM_008258230.1	AF031526.1	XM_008268230.1	XM_008263024.1	XM_002720984.2	XM_002717186.1	XM_008269901.1
Otolemur_crassicaudatus	AB112591.1		AB112590.1					
Otolemur_garnettii	XM_003796229.2	XM_006504966.2	XM_003802556.1	XM_003800078.2	XM_003789251.2	XM_003783729.2	XM_003798152.2	XM_008953547.1
Pan_paniscus	XM_003829435.2	AH005812.2		XM_003824550.2	XM_003833187.1	XM_008953557.1	XM_003830020.2	XM_009458846.1
Pan_troglodytes	XM_516740.5	NM_001009127.1	XM_003317779.1	XM_514302.5	XM_009451417.1	XM_009458833.1	XM_001138878.3	XM_003903672.2
Papio_anubis	XM_003906878.2	XM_003896561.2	NM_001168797.1	XM_009196635.1	XM_009205336.1	XM_009214476.1	XM_003899089.2	XM_006976740.1
Peromyscus_maniculatus	XM_006978532.1	XM_006979426.1	XM_006992450.1	XM_006996184.1	XM_006994448.1	XM_006976683.1	XM_006970386.1	AY726733.1
Phodopus_sungorus		AY029604.1						
Pongo_abelii	XM_002813145.3	XM_002818421.2	XM_002832301.1	XM_002809252.3	XM_002816969.1	XM_009245403.1	XM_002815057.2	XM_009245400.1

Species	*RH1*	*OPN1sw1*	*OPN1lw*	*OPN3*	*OPN5*	*RGR*	*RRH*	*OPN4m*
Propithecus_coquereli	XM_012655172.1	XM_012664772.1	XM_012662045.1	XM_012644391.1	XM_012664670.1	XM_012648008.1	XM_012655079.1	XM_012651274.1
Rattus_norvegicus	NM_033441.1	NM_031015.1	NM_053548.1	NM_001191933.1	NM_181772.1	XM_008771101.1	NM_001107726.1	NM_138860.1
Rhinopithecus_roxellana	XM_010381505.1	XM_010378740.1	XM_010373544.1	XM_010384714.1	XM_010362833.1	XM_010376335.1	XM_010389068.1	XM_010380490.1
Saguinus_mystax			AH006288.2					
Saimiri_boliviensis	XM_003926159.2	XM_003921010.2	XM_003943902.1	XM_010345874.1	XM_003923093.2	XM_010330400.1	XM_003929459.1	XM_003929949.2
Saimiri_sciureus			AH006282.2					
Sciurus_carolinensis		DQ302163.1	AF132044.1					
Spermophilus_tridecemlineatus	XM_005333784.1	XM_005319430.1	NM_001282263.1	XM_005326574.1	XM_005318596.1	XM_005340085.1	XM_005330094.1	XM_005340562.1
Tarsius_syrichta	XM_008047820.1	XM_008063590.1	XM_008071543.1	XM_008063894.1	XM_008062681.1	XM_008067019.1	XM_008065518.1	XM_008060099.1
Tupaia_chinensis	XM_006160664.1	XM_006146852.1	XM_006166677.1	XM_006142367.1	XM_006149093.1	XM_006148709.1	XM_006162245.1	XM_006165495.1
