# Supplementary material for: Gene loss, adaptive evolution and the co-evolution of plumage coloration genes with opsins in birds
Source: BMC Genomics. 2015 Oct 6;16:751. doi: 10.1186/s12864-015-1924-3 (PMC4595237; doi:10.1186/s12864-015-1924-3)
Supplement: Additional file 14: — Saturation analysis. The presence of saturation in base substitution for each of the gene alignment was tested by comparing half of the theoretical saturation index expected when assuming full saturation (Iss.c, critical value) with the observed saturation index (Iss). Absence of substitution saturation is verified when Iss is lower than Iss.c for a significant p-value. (PDF 24 kb) [file 12864_2015_1924_MOESM14_ESM.pdf]

A. Analysis for the avian genes

	Iss	Iss.c	t	df	p-value
RH1	0.141	0.743	55.584	935	0.000
RH2	0.118	0.752	59.840	1064	0.000
OPN1sw1	0.118	0.752	59.464	1064	0.000
OPN1sw2	0.242	0.766	30.103	1022	0.000
OPN1lw	0.302	0.762	40.769	956	0.000
OPN4x	0.207	0.761	55.063	1205	0.000
OPN4m	0.131	0.748	74.345	1004	0.000
OPN3	0.082	0.708	60.807	551	0.000
TMT2	0.357	0.710	14.007	575	0.000
TMT	0.132	0.725	40.512	566	0.000
PIN	0.321	0.751	34.114	1052	0.000
VA	0.207	0.743	41.509	929	0.000
RGR	0.205	0.739	34.344	884	0.000
RRH	0.134	0.741	65.866	911	0.000
OPN5	0.222	0.768	40.788	1070	0.000
MC1R	0.136	0.738	46.713	872	0.000
TYR	0.163	0.778	71.701	1586	0.000
TYRP1	0.113	0.769	71.390	1370	0.000
OCA2	0.173	0.791	90.704	2270	0.000
ASIP	0.241	0.693	20.089	416	0.000

B. Analysis for the mammalian opsins

	Iss	Iss.c	t	df	p-value
RH1	0.284	0.751	36.203	1043	0.000
OPN1sw1	0.307	0.751	34.954	1043	0.000
OPN1lw	0.340	0.754	27.853	1088	0.000
OPN4m	0.280	0.770	38.115	1391	0.000
OPN3	0.392	0.761	24.914	1202	0.000
RGR	0.236	0.738	33.464	872	0.000
RRH	0.276	0.749	40.882	1010	0.000
OPN5	0.189	0.752	52.204	1055	0.000
